# Unravelling Egyptian blue Lily (*Nymphaea nouchali*) organs’ metabolome via *UHPLC/PDA/ESI-QTOF-MS* and in relation to their antioxidant and anti-cholinesterase effects

**DOI:** 10.1038/s41598-025-30937-y

**Published:** 2025-12-19

**Authors:** Inas Y. Younis, Ahmed F. Essa, Samah A. El-Newary, Abdelbaset M. Elgamal, Mohamed A. Farag, Engy Mohsen

**Affiliations:** 1 Pharmacognosy Department, Faculty of Pharmacy, Cairo University, Cairo, 11562 Egypt; 2 Chemistry of Natural Compounds Department, National Research Centre, 33 El-Bohouth St., Dokki, Giza, 12622 Egypt; 3 Pharmaceutical Industries Research Division, Medicinal and Aromatic Plants Research Department, National Research Centre, El-Bouhoths St., Dokki, Cairo, 12622 Egypt; 4 Department of Chemistry of Microbial and Natural Products, National Research Centre, 33 El-Bohouth St., Dokki, Cairo, 12622 Egypt; 5 Health Care Faculty, Saxony Egypt University (SEU), Badr City, Egypt

**Keywords:** Nymphaea, Blue water lily, UHPLC/PDA/ESI-MS, ellagitannins, Lipids, Anti-cholinesterase activity, Biochemistry, Biological techniques, Chemical biology, Chemistry, Drug discovery, Plant sciences

## Abstract

*Nymphaea nouchali* is a prevalent water lily well recognized for its dietary and traditional medicinal purposes, attributed to its diverse phytochemical composition. This study is the first to provide a comprehensive metabolite profiling of Egyptian *Nymphaea* organs viz., flower, leaf, and stem using *UHPLC/PDA/ESI-QTOF-MS* high-resolution ultra-performance liquid chromatography Electrospray ionization/ photo diode array detector/ Quadrupole time-of-flight mass spectrometry. Alongside the in-vitro antioxidant activities employing different methods viz., DPPH, ATBS, NO scavenging, hydrogen peroxide scavenging, and cholinesterase inhibitory (AChE) activity were investigated. A total of 185 secondary metabolites were annotated belonging to 10 chemical classes viz., phenolic acids, ellagitannins, flavonoids, anthocyanins, alkaloids, amino acids and vitamins, organic acids, fatty acids & amides, lipids and sugars/sugar derivatives. Among them, 72 are newly reported viz., ellagic acid, corilagin, patuletin glycosides, naringenin, eriodictyol glycosides, spermidine alkaloids, and 33 lipids in Egyptian *Nymphaea.* Flowers recorded the highest antioxidant activities with IC_50_ values (45.15 µg/ml) for DPPH, (5.02 µg/ml) for ABTS^+^, (54.07 µg/ml) for NO, (55.04 µg/ml) for H_2_O_2_ assays and (1.85 µg/ml) for AChE inhibition. Flowers demonstrated potential antioxidant activities in correlation to their flavonoids, anthocyanins and alkaloids. Multivariate analysis (MVA), including unsupervised tools like principal component analysis (PCA) & hierarchical cluster analysis (HCA), and supervised orthogonal projections to latent structures discriminant analysis (OPLS-DA) are likewise employed. This research is designed to deal in depth with the chemical profile of the Egyptian water lily and tried to correlate such profile with their corresponding neuroprotective effect. Furthermore, in-vivo biological studies are planned to confirm the active agent post isolation from the active organs following results based on chemometric analysis. As future work, it is highlighted to target the isolation of novel entities.

**Supplementary Information:**

The online version contains supplementary material available at 10.1038/s41598-025-30937-y.

## Introduction

The *Nymphaea* genus is a cosmopolitan aquatic plant with a diverse floral morphology. The genus comprises about 50 species belonging to the water-lily family. It is widely distributed in the temperate and tropical regions mainly South Africa along with Nile River^1^. African *Nymphaea* species are designated by the International Union of Conservation of Nature (IUCN) in the Red-List and subjected to significant geographical variations due to climate changes attributed to the global warming^2^. Since ancient times, water lilies symbolize more than spiritual significance, being included as a food in several dishes^3,4^. Moreover, in ancient Egyptian, three species of water-lily were directly associated with the Egyptian civilization viz.: *N. lotus* (white lotus), *N. Speciosum* (pink lotus) and *N. caerulea* ( blue lotus), regarded as a sacred symbol of Egyptian life^5^.

Among *Nymphaea* taxa, *N. nouchali* Burm. var.*caerulea* has been recently reported in the Flora of Egypt as an ancient treasure of food and medicine^6,7^. The magnificent flowers of *N. caerulea* bloom in September in a star-like pattern with a range of colors, as blue, white, and pink, with blue being the most popular^8^.

Moreover, flowers were not only utilized as a natural food colorant but also served as an integral component in functional beverages, particularly when paired with green tea^9^. Importantly, leaves, stems and seeds are used as a staple food with a superior content of amino acids especially glutathione the primary precursor of neurotransmitter (γ amino butyric acid)^10^. Likewise, flowers and leaves exhibit potential antioxidant, antihyperglycemic, and antihyperlipidemic activities^11^.

Several polyphenolics were identified in *N. nouchali* stem collected from Bangladesh with an antioxidant activity^12^. Recently^4,8^ documented the presence of many bioactive compounds as flavonoids, anthocyanins, coumarins, tannins, fatty acids, and anthraquinones in the Chinese blue flowers.

It is well established that an imbalance between the endogenous and exogenous antioxidant mechanisms leads to the generation of free radicals including reactive oxygen species (ROS). The later reacts with biological macromolecules such as proteins, lipids, and cellular DNA, and eventually leads to brain oxidative damage, as in case of Alzheimer’s disease (AD) associated with the deposition of A*β* plaque^13^. Gallic acid the primary bioactive metabolite of Indian *N. pubescens* flowers exhibited promising anticholinesterase and antioxidant activities^14^. Furthermore, phenolic compounds, such as flavonoids and tannins, have been shown to mitigate oxidative stress and offer protective effects against cellular damage^15,16^.

Alzheimer’s disease (AD) is a specific form of adult-onset dementia that is clinically associated with a gradual degeneration of the structure and function of the specific brain neurons. Globally, the estimated number of dementia will increase dramatically to reach 152 million cases in 2050 according to the recent World Alzheimer Report 2019 with high rates of comorbidity compared to other chronic disorders^17^. Extracellular plaque deposition of the *β*-amyloid peptide (A*β*) and hyperphosphorylation of tau protein are the primary hallmark of AD associated with rapid decline of acetylcholine level along with oxidative damage. For the past 30 years, “amyloid hypothesis” was the only accepted mainstream theory for AD; however, it failed to improve the cognitive outcomes^15,16^. Recently, the therapeutic strategy was based on improvement of the impaired cholinergic neurotransmission by inhibition of the degradation of acetylcholinesterase (AChE). Currently, donepezil, the classical AChE inhibitor that FDA approved as a classical drug for the symptomatic management of mild AD. Unfortunately, bradycardia, anorexia and gastrointestinal symptoms with high mortality rate, were observed as adverse effects of donepezil especially in elder patients^18^.

Ultra high-performance LC coupled to high-resolution MS (UHPLC/HR-MS/MS) is usually employed for the untargeted analysis of the complex structure of plant-based metabolites^19^. *UHPLC/PDA/ESI-QTOF-MS* coupled with the Global Natural Products Social Molecular Networking (GNPS) platform aids in high throughput annotation of plant secondary metabolites. GNPS as a computational visualization technique was able to group compounds according to their similarities in MS/MS spectra and successfully uncover the connections between several analogues^20^.

The classification of water lily organs was established through *UHPLC/PDA/ESI-QTOF-MS* analysis coupled with molecular networking along with multivariate data analysis to allow the characterization of markers for the tested specimens. PCA (principal component analysis) was initially constructed to reveal variation among water lily organs followed by OPLS-DA (orthogonal partial least squares-discriminant analysis) to emphasize PCA results. OPLS model derived from UPLC-MS was more predictive for organs´ classification and identification of markers for each organ.

The present study aimed to provide comprehensive metabolites profiling of different organs viz., flower, leaf and stem in *Nymphaea nouchali var. caerulea via UHPLC/PDA/ESI-QTOF-MS* for the first time alongside with their in vitro antioxidant and anticholinesterase effects. This approach not only provides valuable insights into *N. nouchali* bioactive constituents but rather highlights the potential uses of different organs based on such a comprehensive approach revealing its potential in development of health-promoting foods.

## Materials and methods

### Plant material

Fresh plants of Egyptian *N. nucifera* (flowers, leaves, and stems) were collected from El-Orman botanical garden in August 2021 after the necessary permission was obtained from “Agricultural Research Center, Giza, Egypt” in “9, Cairo University Road, Oula, Giza District, Giza Governorate”. It was kindly authenticated by Professor Dr. Mohamed Gebaly, National Research Center, Giza, Egypt. A voucher specimen was deposited at the Herbarium of the Pharmacognosy Department, Faculty of Pharmacy, Cairo University, Egypt under Herbarium No. (3-8-2021).

### Chemicals and materials

All chemicals and reference standards were purchased from Sigma Aldrich, USA. Screening Kit of acetylcholinesterase inhibitor was obtained from Quanti Chrom TM, USA.

### Determination of total phenolic, flavonoids and tannins contents

The total phenolic and flavonoids contents were accurately determined according to^21^ and were expressed as mg equivalent gallic acid and mg equivalent of quercetin/g of extract respectively. Meanwhile, total tannin content was estimated using Folin–Ciocalteau’s reagent according to^22^.

### Preparation of Lotus extracts for UHPLC/PDA/ESI-QTOF-MS analysis

The air-dried flowers, leaves, and stems (100 g each) were pulverized into a fine powder using liquid nitrogen. The fine powder of each organ (20 mg) was mixed with 2 ml 80% methanol containing 10 µg/mL umbelliferone as an internal standard then they were homogenized using an Ultra-Turrax mixer (11,000 rpm, IKA, Staufen, Germany) for 5 × 60 s periods with 1 min intervals according to previous reported method^23^. Sample extracts are stored at -40 °C.

### High-resolution ultra-performance liquid chromatography-mass spectrometry analysis (UHPLC/PDA/ESI-qTOF-MS)

Acquity Ultra-Performance Liquid Chromatograph (UPLC, Waters) coupled to a photo diode array detector (PDA, Waters) and an electrospray ionization quadrupole time-of-flight tandem mass spectrometer, (Waters, Milford, MA) were employed for the separation. HSS T3 column (100 × 1.0 mm, particle size 1.8 μm; Waters) was established to perform the chromatographic separation according to the method previously described by^24^.

### Molecular networking in both negative and positive modes

The raw data files generated by ESI–QTOF–MS/MS in both acquisition modes were converted to mzXML format *via* the MS Convert tool from ProteoWizard (version 3.0.21050, available at https://proteowizard.org). The converted files were subsequently uploaded to the MassIVE repository via WinSCP (https://massive.ucsd.edu). The classical molecular networking (MN) of the LC-QTOF-MS/MS data was conducted using the Global Natural Product Social (GNPS) MN platform. With the default GNPS networking parameters, the analysis was performed with a precursor ion mass tolerance of 0.2 Da and a fragment ion tolerance of 0.05 Da. Not less than 6 matching MS fragments between 2 consensus MS/MS spectra were required, with a cosine similarity score of 0.7 or greater to establish connections between nodes. The job at negative and positive modes on GNPS can be found at (https://gnps.ucsd.edu/ProteoSAFe/status.jsp?task=d845b50ccecb4d508b803b5f7ad2ec91) and (https://gnps.ucsd.edu/ProteoSAFe/status.jsp?task=f7613c61045f4de482b93a94f85c81f6) respectively. The graphML file of resulting network was exported as a and visualized using a force-directed layout in Cytoscape 3.7.1as previously described^24^.

### In vitro antioxidant assays

To evaluate the antioxidant potential of *Nymphaea* extract, several in vitro assays viz.: DPPH, ABTS, hydrogen peroxide scavenging, NO scavenging, and metal chelation were employed. Five concentrations from each extract were prepared in 100% methanol at 1000, 500, 250, 125, and 62.50 µg/ml. The antioxidant activity of *Nymphaea* organ extracts was evaluated compared with L-ascorbic acid (natural antioxidant) and Trolox (synthetic antioxidant) prepared under similar conditions. All absorbances were measured spectrophotometrically using (Jasco, serial No. C317961148, Japan).

#### DPPH assay

Free radical scavenging was assessed using DPPH (2,2-diphenyl-1-picrylhydrazyl) as previously described^25^. Absorbance was recorded using UV Spectrophotometer (Jasco, serial No. C317961148, Japan) set at 517 nm for triplicate assays for each sample.

#### ATBS assay

The antioxidant activity was evaluated in triplicate according to^16,26^. The stable blue-green of the cationic chromophore 2,2-azinobis-(3-ethylbenzothiazoline-6-sulfonate) (ABTS^•+^) was measured at 734 nm using UV Spectrophotometer (Jasco, serial No. C317961148, Japan).

#### Hydrogen peroxide scavenging assay

The method of^16^ was used to assess *Nymphaea* extracts’ capacity to scavenge hydrogen peroxide(H_2_O_2_). The absorbance of reaction mixture was determined at 230 nm against a blank solution without hydrogen peroxide using UV Spectrophotometer (Jasco, serial No. C317961148, Japan).

#### NO scavenging assay

Sodium nitroprusside (SNP) generating nitric oxide NO^•^ system was used for measuring the ability of *Nymphaea* extracts to scavenge NO^•^ following exact procedure described in^16^. Under physiological pH, the generated NO^•^ reacted with oxygen to develop nitrite ions that measured by the Greiss reagent. The absorbance of each solution was measured at 540 nm against the corresponding blank solution UV Spectrophotometer (Jasco, serial No. C317961148, Japan).

### Determination of anti-cholinesterase activity

The cholinesterase inhibitory activity (AChE) was evaluated using the Quanti Chrom™ Acetylcholinesterase Screening Kit (BioAssay Systems, Hayward, CA, USA), following the method of^27^ compared to Donepezil as a standard cholinesterase inhibitor. The calibration curves were constructed by plotting % inhibition against the logarithm of inhibitor concentration (0, 0.1, 1, 10 µg/ml) of each extract. IC_50_ values were determined using log-probit analysis according to the following equation:

$$\% {\text{ }}Enzyme\;inhibition={\text{ }}\left( {E - S/E * 100} \right).$$

Where E is the enzyme activity without extract and S is the activity of enzyme in the presence of the extract. The assay was performed in triplicate using descriptive statistics.

### Statistical analysis

Statistical analysis of each sample was performed in triplicate and expressed as mean ± SD using one-way ANOVA followed by the Tukey-Kramer test using Graph Pad prism version 9 (GraphPad, San Diego, CA). The results were considered statistically significant at *p* < 0.05.

## Results and discussion

### Determination of total phenolics, flavonoids and tannins contents

Total phenolics (TP), flavonoids (TF) and tannins (T) assays revealed that flower extract exhibited the highest levels of TP (561.5 ± 15.1 mg/g) extract, TF (116.113 ± 7.69 mg/g) extract and T( 413.952 ± 7.13 mg/g). Unfortunately, stem extract displayed the lowest content 324.83 ± 6.83 mg/g extract, 41.17 ± 1.72 mg/g extract and 277.57 ± 12.91 mg/g extract for TP, TF and T respectively Suppl. Fig S1.

### UHPLC/PDA/ESI-QTOF-MS based metabolites profiling of *N. nouchali* organs with molecular networking

**Fig. 1:**
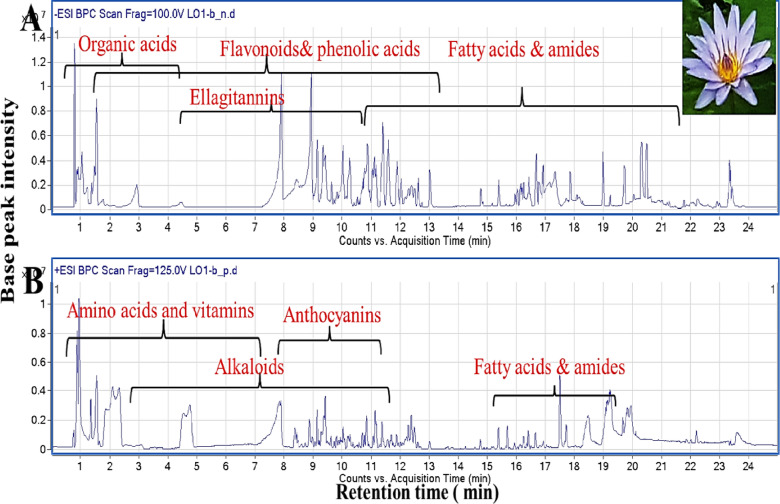
UPLC-qTOF-MS base peak chromatogram of *Nymphaea caerulea flowers* ethanolic extract detected in (**A**) negative and (**B**) positive ionization modes. Peak numbers follow that listed in Table 1 for metabolites identification using UPLC-PDA-MS.

To identify the exact chemical composition in different organs, UHPLC/PDA/ESI-QTOF-MS was used for profiling Egyptian water lily different organs, Fig. 1, Suppl. Figs. S2, S3. Metabolites identification was based on retention time, molecular formula, fragmentation pattern obtained in both ionization modes (negative and positive) and compared to previous literature as listed in Table 1. A total of 185 metabolites were annotated belonging to phenolic acids, ellagitannins, flavonoids, anthocyanins, alkaloids, amino acids, vitamins, and lipids. To the best of our knowledge, this is the first report of untargeted metabolite profiling in Egyptian *N. caerulea* coupled with GNPS molecular networking and chemometrics to investigate variations in both primary and secondary metabolites among organs.

**Table 1:** Characterization of the chemical constituents of *Nymphaea Nouchali* var. Caerulea using UPLC-HR-ESI-MS/MS in negative and positive modes (*n* = 3).

Peak no.	Rt (min)	Molecular ion (m/z) (M + H)^−^/(M + H)^+^	Error (ppm)	Ionic Formula	Putative Identification	MS/MS (m/z)	Fl	St	L
*Phenolic acid*									
1	1.510	405.0252	-5.65	C_21_H_9_O_9_^−^	Gallic acid derivative	169,125	+	+	+
2	2.734	169.0142	1.45	C_7_H_5_O_5_^−^	Gallic acid	125	+	+	+
3	4.078	483.078	3.57	C_20_H_19_O_14_^−^	Di-galloyl-*O*- hexose	331, 169	+	+	+
4	4.768	331.0671	3.83	C_13_H_15_O_10_^−^	Galloyl-*O*-hexose	169,125	+	+	+
5	5.884	495.078	4.09	C_21_H_19_O_14_^−^	Di galloyl-*O*-quinic acid	301,169	+	-	+
6	6.804	315.0722	1.76	C_13_H_15_O_9_^−^	Protocatechuoyl-*O*-hexoside	153	+	+	-
7	7.817	373.0776	1.96	C_15_H_17_O_11_^−^	Acetyl-*O*-galloyl hexose	169, 125	+	+	+
8	7.985	299.0772	3.47	C_13_H_15_O_8_^−^	Salicylic acid-*O*- hexoside	137	+	+	+
9	8.105	325.0138	5.84	C_14_H_13_O_9_^−^	Galloyl shikimic acid	169	+	+	+
10	8.718	329.0878	2.44	C_14_H_17_O_9_^−^	Vanillic-*O*-hexoside	167	+	+	+
11	8.883	183.0299	1.61	C_8_H_7_O_5_^−^	Methyl-*O*- gallate	125	+	+	+
12	8.870	153.0193	2.16	C_7_H_5_O_4_^−^	Protocatechuic acid	135,109	+	+	+
13	8.997	295.0459	2.84	C_13_H_11_O_8_^−^	Caffeoyl malic acid	191	+	+	+
14	9.232	236.0564	1.88	C_11_H_10_NO_5_^−^	Benzoyl aspartic acid	192, 121	+	+	-
15	9.456	477.0675	3.48	C_21_H_17_O_13_^−^	Di-*O*-galloylshikimic acid	289, 169	+	+	+
16	9.062	291.0146	3.56	C_13_H_7_O_8_^−^	Brevifolin carboxylic acid	247, 219, 191,175	+	+	+
17	9.444	355.1035	2.4	C_16_H_19_O_9_^−^	Feruloyl-*O*- hexoside	193	+	+	+
18	9.479	385.114	2.9	C_17_H_21_O_10_^−^	Sinapoyl -*O*- hexoside	223, 205,190	+	-	+
19	9.518	605.0784	3.5	C_26_H_21_O_17_^−^	Galloyl brevifolincarboxyl hexoside	169	+	+	+
20	9.566	353.0514	3.16	C_15_H_13_O_10_^−^	Caffeoyl citrate	191	+	+	+
21	9.632	321.0252	2.19	C_14_H_9_O_9_^−^	Digallic acid	169,125	+	+	+
22	10.052	305.0303	3.89	C_14_H_9_O_8_^−^	Methyl brevifolincarboxylate	247	+	+	+
23	10.448	193.0506	2.74	C_10_H_9_O_4_^−^	Ferulic acid	134	+	-	+
*Ellagitannins*									
24	1.635	481.0624	0.16	C_20_H_17_O_14_^−^	Ellagic acid derivative	300.99	+	-	+
25	5.879	495.078	0.86	C_21_H_19_O_14_^−^	Di-*O*-galloyl quinic acid	191,169	+	-	+
26	7.954	455.177	3.1	C_18_H_31_O_13_^−^	Ellagic acid derivative	301	+	+	+
27	8.534	633.0733	2.42	C_27_H_21_O_18_^−^	Corilagin	301,169	+	+	+
28	8.892	785.0843	3.3	C_34_H_25_O_22_^−^	Tellimagradin I	483, 301, 169	-	-	+
29	8.962	983.1946	-1.9	C_41_H_43_O_28_^−^	Corilagin derivative	633, 301	+	+	+
30	9.007	801.0792	3.13	C_34_H_25_O_23_^−^	Punigluconin	757, 301	+	+	+
31	9.275	785.0843	3.3	C_34_H_25_O_22_^−^	Di-*O*-galloyl Hexahydroxy diphenoyl -glucopyranoside	633, 300.99, 169	+	+	+
32	9.296	786.0862	1.59	C_41_H_22_O_17_^−^	Ellagic acid derivative	301	-	-	+
33	9.345	783.0675	0.57	C_34_H_23_O_22_^+^	Pedunculagin	463,303	-	+	+
34	9.444	951.0686	-2.79	C_48_H_23_O_22_^−^	Geraniin	463,301,169	+	+	-
35	9.553	463.0518	4.12	C_20_H_15_O_13_^−^	Ellagic-*O*-hexoside	300.99	+	+	+
36	9.603	635.089	2.81	C_27_H_23_O_18_^−^	Trigalloyl hexose	465,300.99, 165	+	+	+
37	9.618	925.0953	3.08	C_40_H_29_O_26_^−^	Ellagic acid derivative	755,300.99, 169	+	+	+
38	9.665	801.0781	2.02	C_34_H_25_O_23_^+^	Lagerstannin A	463, 303, 277	+	+	+
39	9.728	935.0796	2.78	C_41_H_27_O_26_^−^	Casuarictin	633, 300.99	+	+	-
40	9.915	491.0467	2.9	C_21_H_15_O_14_^−^	Ellagic acid derivative	300.99,169	+	+	+
41	9.949	787.0999	4.12	C_34_H_27_O_22_^−^	Tetra-*O*-galloyl-hexose	635, 465, 169	+	+	+
42	9.965	433.0412	4.26	C_19_H_13_O_12_^−^	Ellagic acid-*O*-pentoside	301	+	+	+
43	10.017	965.0902	3.38	C_42_H_29_O_27_^−^	Castalagin derivative	633, 463, 301	+	+	+
44	10.226	300.999	3.28	C_14_H_5_O_8_^−^	Ellagic acid	283,257,229	+	+	+
45	10.23	765.0945	2.96	C_35_H_25_O_20_^−^	Methyl-*O*–di-*O*-galloyl-rhamnopyranosyl ellagic acid	463, 301	-	+	+
46	10.293	939.1109	4.05	C_41_H_31_O_26_^−^	Penta galloyl hexose	301, 191	+	+	-
47	10.509	997.1164	4.89	C_43_H_33_O_28_^−^	Punicatannin A	464,300.99, 169	+	+	+
48	10.518	997.1164	2.99	C_43_H_33_O_28_^−^	Punictannin	633, 463, 300.99, 169	+	-	+
49	10.936	953.0902	3.43	C_41_H_29_O_27_^−^	Chebulagic acid	935,617, 301, 160	+	-	-
*Flavonol*									
50	7.760	933.3398	5.1	C_38_H_61_O_26_^−^	Quercetin hexosyl hexosyl pentosyl glucuronic acid	477, 301	+	-	+
51	8.357	364.1364	0.55	C_15_H_24_O_10_^+^	Quercetin-derivative	303	+	-	+
52	8.985	617.1137	1.15	C_28_H_25_O_16_^+^	Quercetin galloyl hexoside	303	+	+	+
53	9.186	601.1188	4.16	C_28_H_25_O_15_^+^	Kaempferol galloyl hexoside	287	-	+	-
54	9.599	611.1607	0.1	C_27_H_31_O_16_^+^	Kaempferol-di-*O*-hexoside	449,287	+	+	+
55	9.649	609.1461	3.62	C_27_H_29_O_16_^−^	Quercetin -*O*-rhamnoside-*O*-hexoside	447, 301	+	-	-
56	9.792	449.1078	-1.25	C_21_H_21_O_11_^+^	Kaempferol-*O*-hexoside	287	+	+	+
57	9.876	479.0831	2.95	C_21_H_19_O_13_^−^	Myricetin- *O*-hexoside	316	+	+	+
58	9.998	631.0941	3.6	C_28_H_23_O_17_^−^	Myricetin galloyl hexoside	317,179	+	+	+
59	10.108	463.0882	2.15	C_21_H_19_O_12_^−^	Myricetin-*O*- rhamnoside	316	+	+	+
60	10.155	449.0725	4.55	C_20_H_17_O_12_^−^	Myricetin-*O*-pentoside	316	+	+	-
61	10.202	319.0448	0.14	C_15_H_11_O_8_^+^	Myricetin	273, 179	+	+	+
62	10.302	647.1243	3.06	C_29_H_27_O_17_^+^	Patuletin galloyl hexoside	333	-	+	-
63	10.312	487.0871	4.94	C_23_H_19_O_12_^+^	Quercetin derivative	303	-	+	+
64	10.404	615.0992	3.83	C_28_H_23_O_16_^−^	Quercetin-*O*-galloyl-hexoside	301	+	+	+
65	10.418	749.0621	1.16	C_34_H_21_O_20_^+^	Quercetin derivative	303	-	+	+
66	10.612	495.1133	1.65	C_22_H_23_O_13_^+^	Patuletin- *O*-hexoside	333	-	+	+
67	10.641	643.1294	1.03	C_30_H_27_O_16_^+^	Myricetin derivative	319	-	-	+
68	10.675	447.0933	3.76	C_21_H_19_O_11_^−^	Quercetin-*O*-rhamnoside	301	+	-	-
69	10.689	303.0499	0.1	C_15_H_11_O_7_^+^	Quercetin	151	+	+	+
70	10.777	505.0969	3.29	C_23_H_21_O_13_^−^	Myricetin*-O-*acetyl rhamnoside	316	-	-	+
71	10.79	435.0922	0.89	C_20_H_19_O_11_^+^	Quercetin- *O*-pentoside	303	-	+	+
72	10.895	319.0442	2.02	C_15_H_11_O_8_^+^	Myricetin isomer	273,179	+	+	+
73	10.919	465.1028	0.33	C_21_H_21_O_12_^+^	Patuletin-*O*-rhamnoside	333	+	+	+
74	10.940	463.0882	4.52	C_21_H_19_O_12_^−^	Quercetin-*O*- hexoside	301	+	+	+
75	11.007	505.0988	4.28	C_23_H_21_O_13_^−^	Myricetin*-O-*acetyl rhamnoside isomer	316	-	-	+
76	11.13	615.0992	3.99	C_28_H_23_O_16_^−^	Patuletin galloyl pentoside	331	-	+	+
77	11.166	625.1199	2.55	C_30_H_25_O_15_^−^	Myricetin*-O-*coumaroyl hexoside	479, 317/316	+	+	+
78	11.164	625.1199	4.46	C_30_H_25_O_15_^−^	Nympholide A	479, 317,179	+	+	+
79	11.207	626.1218	0.39	C_37_H_22_O_10_^−^	Myricetin derivative	317	-	+	+
80	11.23	461.1089	3.54	C_22_H_21_O_11_^−^	Isorhamnetin-*O*-rhamnoside	314/315	+	-	-
81	11.284	477.1402	4.47	C_23_H_25_O_11_^−^	Isorhamnetin-*O*-hexoside	315	+	+	+
82	11.359	625.1199	3.34	C_30_H_25_O_15_^−^	Myricetin*-O-*coumaroyl hexoside isomer	317/316	+	+	+
83	11.386	331.0459	1.33	C_16_H_11_O_8_^−^	Methyl myricetin	316	+	+	+
84	11.389	331.0459	1.33	C_16_H_11_O_8_^−^	Patuletin	301	+	+	+
85	11.614	489.1038	3.77	C_23_H_21_O_12_^−^	Quercetin -*O*-acetyl- rhamnoside	301	+	+	+
86	11.646	639.1355	3.97	C_31_H_27_O_15_^−^	Quercetin-feruloyl hexoside	463,301	-	+	+
87	11.874	947.2158	-7.63	C_35_H_47_O_30_^−^	Kaempferol derivative	284	+	-	-
88	11.897	473.1089	3.24	C_23_H_21_O_11_^−^	Kaempferol-*O*-acetyl-rhamnoside	285/284	+	+	+
89	11.924	653.1512	4.42	C_32_H_29_O_15_^−^	Isorhamnetin-*O*-feruloyl hexoside	477,315	+	+	-
90	11.961	503.1195	3.57	C_24_H_23_O_12_^−^	Isorhamnetin*-O-*derivative	315	+	-	-
91	12.183	315.051	0.4	C_16_H_11_O_7_^−^	Methyl quercetin	301	+	+	+
92	12.726	531.1144	2.66	C_25_H_23_O_13_^−^	Quercetin*-O-*derivative	301	+	-	-
93	13.233	515.1195	3.1	C_25_H_23_O_12_^−^	Diacetyl kaempferol-*O*- rhamnoside	284/285	+	+	-
94	14.688	477.2494	0.61	C_26_H_37_O_8_^−^	Isorhamnetin-*O*-derivative	315	+	+	+
95	15.118	593.2662	-6.69	C_23_H_45_O_17_^−^	Isorhamnetin*-O-*derivative	315	+	+	+
96	15.814	663.2717	-5.11	C_26_H_47_O_19_^−^	Isorhamnetin*-O-*derivative	315	-	+	+
*Flavone*									
97	8.989	477.1614	2.85	C_20_H_29_O_13_^−^	Apigenin hexoside derivative	431, 269	+	-	-
98	9.306	449.1087	-0.37	C_21_H_21_O_11_^−^	Apigenin derivative	269	+	-	-
99	9.668	563.1406	3.42	C_26_H_27_O_14_^−^	Apigenin-*C*-hexoside-*C*-arabinoside	503, 473, 443, 383	-	+	-
*Flavanones*									
100	10.141	433.1129	5.81	C_21_H_21_O_10_^−^	Naringenin-*O*-hexoside	271	-	+	-
101	10.575	595.1668	0.24	C_27_H_31_O_15_^−^	Eriodictyol *O*-hexoside *C*-hexoside	301,475, 271	+	+	+
102	10.555	435.1297	3.37	C_21_H_23_O_10_^−^	Phloretin *O*-hexoside (Phlorizin)	273, 167	+	+	+
103	10.853	449.1089	2.97	C_21_H_21_O_11_^−^	Eriodictyol-*O-*hexoside	287	+	-	-
104	11.076	477.1402	5.72	C_23_H_25_O_11_^−^	Naringenin*-O-*derivative	271	+	+	+
105	11.176	433.114	2.58	C_21_H_21_O_10_^−^	Naringenin-*O*-hexoside isomer	271	+	-	-
106	11.544	431.1348	3.83	C_22_H_23_O_9_^−^	Naringenin*-O-* derivative	271	+	+	+
107	12.522	271.0612	3.3	C_15_H_11_O_5_^−^	Naringenin	177, 151,119	+	-	-
*Anthocyanins*									
108	8.908	627.1556	0.44	C_27_H_31_O_17_^+^	Delphinidin*-O-* derivative	481, 319,303	+	-	-
109	9.451	507.1133	2.6	C_23_H_23_O_13_^+^	Delphinidin*-O-* acetyl hexoside	465,303	+	-	-
110	9.833	796.0882	,0.16	C_37_H_25_O_19_ Na^+^	Delphinidin*-O-* derivative	303	+	-	-
111	9.972	797.0843	1.37	C_35_H_25_O_22_^+^	Delphinidin*-O-* derivative	303	+	-	-
112	10.303	465.1028	0.33	C_21_H_21_O_12_^+^	Delphinidin*-O-*hexoside	303	+	-	-
113	10.371	815.0949	2.77	C_35_H_27_O_23_^+^	Delphinidin*-O-*derivative	303	+	-	-
114	11.237	479.1184	3.56	C_22_H_23_O_12_^+^	Petunidin*-O-*hexoside	317	+	-	-
*Alkaloids*									
115	3.064	138.055	4.05	C_7_H_8_NO_2_^+^	Trigonelline	97,96	+	+	+
116	10.125	438.2387	-0.15	C_25_H_32_N_3_O_4_^+^	Di-*p*-Coumaroyl spermidine	292,147	+	-	-
117	10.266	468.2493	-0.22	C_26_H_34_N_3_O_5_^+^	Feruloyl coumaroyl spermidine	322,147	+	-	-
118	12.190	584.2755	0.19	C_34_H_38_N_3_O_6_^+^	Tri-coumaroyl spermidine	438,292,147	+	-	-
119	12.241	614.2861	1.92	C_35_H_40_N_3_O_7_^+^	Feruloyl dicoumaroyl spermidine	468,292,147	+	+	-
120	12.318	644.2966	0.22	C_36_H_42_N_3_O_8_^+^	Di-feruloyl coumaroyl spermidine	468,147	+	+	-
*Amino acids and vitamins*									
121	1.540	290.0881	4.95	C_11_H_16_NO_8_^−^	Hexosyl pyroglutamic acid	128	+	+	+
122	1.553	128.0353	3.23	C_5_H_6_NO_3_^−^	Pyroglutamic acid	85	+	+	+
123	1.387	123.0553	1.55	C_6_H_7_N_2_O^+^	Nicotinamide	106	+	+	+
124	3.544	177.1022	-0.34	C_10_H_13_N_2_O^+^	Hydroxy tyramine	160	+	+	+
125	4.644	166.0863	-4.51	C_9_H_12_NO_2_^+^	Phenylalanine	149,122	+	+	+
126	7.484	220.1179	-1.15	C_9_H_18_NO_5_^+^	Pantothenic acid B5	202 ,184	+	+	+
127	8.334	205.0983	6.07	C_11_H_13_N_2_O_2_^+^	Tryptophan	188,170, 146	+	+	+
128	8.435	298.0968	2.48	C_11_H_16_N_5_O_3_S^+^	Methyl thioadenosine	136	+	+	+
129	8.665	161.1073	0.16	C_10_H_13_N_2_^+^	Tryptamine	144	+	-	-
*Organic acids*									
130	1.033	133.0142	5.57	C_4_H_5_O_5_^−^	Malic acid	115, 72, 71	+	-	-
131	1.280	173.0455	3.14	C_7_H_9_O_5_^−^	Shikimic acid	137	+	+	+
132	1.332	191.0197	2.22	C_6_H_7_O_7_^−^	Citric acid	111, 87, 76	+	+	+
133	1.472	203.0197	-0.36	C_7_H_7_O_7_^−^	Daucic acid	159	+	-	+
134	1.696	161.0455	3.38	C_6_H_9_O_5_^−^	Hydroxy adipic acid	117	+	+	-
135	1.888	117.0193	2.81	C_4_H_5_O_4_^−^	Succinic acid	99	+	+	+
136	5.205	143.035	3.35	C_6_H_7_O_4_^−^	Methylglutaconic acid	99,83	-	+	+
*Fatty acids & amides*									
137	10.973	187.0976	3.1	C_9_H_15_O_4_^−^	Nonanedioic acid	169	+	+	+
138	12.396	199.134	2.84	C_11_H_19_O_3_^−^	Oxo-undecanoic acid	182	-	+	+
139	12.572	327.2177	2.73	C_18_H_31_O_5_^−^	Trihydroxy-octadecadienoic acid	283	+	+	+
140	13.006	329.2333	0.75	C_18_H_33_O_5_^−^	Trihydroxy-octadecaenoic acid	229,211,183	+	+	+
141	14.774	311.2228	3.15	C_18_H_31_O_4_^−^	Octadecenedioic acid	223	+	+	+
142	16.101	293.2122	3.8	C_18_H_29_O_3_^−^	Hydroxy octadecatrienoic acid	275	+	+	+
143	16.907	454.2928	0.48	C_21_H_45_NO_7_P^+^	Palmitoyl-hydroxy-glycerol-phospho-ethanolamine	313	+	+	+
144	17.551	282.2791	1.21	C_18_H_36_NO^+^	Octadecenamide	149,135	+	+	+
145	18.988	271.2279	3.19	C_16_H_31_O_3_^−^	hydroxy palmitic acid	253,225	+	+	+
146	1.961	277.2173	3.61	C_18_H_29_O_2_^−^	Gamma-Linolenic acid	259, 233,191	+	+	+
147	19.641	282.2791	0.5	C_18_H_36_NO^+^	Oleamide	265, 247	+	+	+
148	19.701	279.233	3.76	C_18_H_31_O_2_^−^	Octadecadienoic acid	261	+	+	+
149	22.371	465.301	4.58	C_30_H_41_O_4_^−^	Oleanadienoic acid	421	-	+	-
*Lipids*									
150	13.684	537.3262*	1.4	C_25_H_47_O_9_^−^	MGMG (16:0)	255	+	+	+
151	14.813	667.4350	2.5	C_37_H_64_O_8_P^−^	PA (16:0/18:3)	431,279	+	-	+
152	15.160	593.2710	1.9	C_27_H_46_O_12_P^−^	PI(18:3/0:0)	315, 277, 153	+	+	+
153	15.378	737.3572*	2.5	C_33_H_55_O_15_^−^	Hydroxy DGMG (18:3)	691, 397, 293	-	-	+
154	15.382	721.36270*	2.0	C_33_H_55_O_14_^−^	DGMG (18:3)	675, 397, 277	+	+	+
155	15.538	577.2688	2.09	C_27_H_45_O_11_S^−^	Sulfoquinovosyl monoacyl glycerol (18:3)	225, 80	+	+	+
156	15.660	595.2890	2.0	C_27_H_48_O_12_P^−^	PI(18:2/0:0)	315, 277, 153	+	+	+
157	15.664	474.2610	1.1	C_23_H_41_NO_7_P^−^	PE(18:3/0:0)	277, 196, 153	+	+	+
158	16.377	555.2845	2.62	C_25_H_47_O_11_S^−^	Sulfoquinovosyl monoacyl glycerol (16:0)	277, 225	-	+	-
159	16.064	723.3786*	1.6	C_33_H_57_O_14_^−^	DGMG (18:2)	677, 397, 279	+	+	+
160	16.066	723.3726	0.16	C_34_H_60_O_14_P^−^	Glycophosphoinositol	279	+	+	+
161	16.116	431.2190	0.7	C_21_H_36_O_7_P^−^	PA(18:3)	277,153,79	+	+	+
162	16.221	562.3130	1.7	C_27_H_49_NO_9_P^−^	PC (18:3)	502, 277	+	+	+
163	16.234	559.3110*	0.5	C_27_H_45_O_9_^−^	MGMG (18:3)	277	+	+	+
164	16.361	476.2783	4.53	C_23_H_43_NO_7_P^−^	PE(18:2/0:0)	279, 196	+	+	+
165	16.412	571.2870	1.3	C_25_H_48_O_12_P^−^	PI(16:0/0:0)	315, 255, 153	+	+	+
166	16.516	699.3790*	2.57	C_31_H_57_O_14_^−^	DGMG (16:0)	563, 397, 255	+	+	-
167	16.938	452.2783	3.23	C_21_H_43_NO_7_P^−^	PE(16:0/0:0)	255,196	+	+	+
168	17.091	564.3290	1.1	C_27_H_51_NO_9_P^−^	PC (18:2)	, 279, 504 224	+	+	+
169	17.141	481.2560	0.2	C_22_H_42_O_9_P^−^	PG(16:1/0:0)	253, 153	+	-	+
170	17.227	561.3260*	1.7	C_27_H_47_O_9_^−^	MGMG (18:2)	279	+	+	+
171	17.390	535.3110*	0.5	C_25_H_45_O_9_^−^	MGMG (16:1)	253	-	-	+
172	17.484	433.2350	0.1	C_21_H_38_O_7_P^−^	PA(18:2)	279,153,79	-	+	+
173	17.540	409.2350	0.1	C_19_H_38_O_7_P^−^	PA(16:0)	255,153,79	+	+	+
174	17.592	483.2710	1.5	C22H44O9P^−^	PG(16:0/0:0)	255, 153	+	+	+
175	18.062	540.3290	1.1	C_25_H_51_NO_9_P^−^	PC (16:0)	480, 255	+	+	+
176	18.502	435.2500	1.4	C_21_H_40_O_7_P^−^	PA(18:1)	281, 153, 79	+	+	+
177	18.827	480.3080	1.0	C_23_H_47_NO_7_P^−^	PE(18:0/0:0)	283, 196, 153	+	+	+
178	19.898	511.3050	3.8	C_24_H_48_O_9_P^−^	PG(18:0/0:0)	283, 153	+	-	-
179	20.134	793.5141	4.18	C_41_H_77_O_12_S^−^	Sulfoquinovosyl diacyl glycerol (16:0/16:0)	537, 277, 255,225	-	+	-
180	20.298	695.4630	2.4	C_39_H_68_O_8_P^−^	PA(18:2/18:2)	433,415,279	-	+	+
181	20.368	693.4520	4.3	C_39_H_66_O_8_P^−^	PA(18:2/18:3)	431,433,279, 277	-	+	+
182	22.184	815.4985	6.21	C_43_H_75_O_12_S^−^	Sulfoquinovosyl diacyl glycerol (16:0/18:3)	537, 277, 255,225	+	+	-
183	22.977	505.2550	2.2	C_24_H_42_O_9_P^−^	PG(18:3/0:0)	277, 153	-	-	+
*Sugars/sugar alcohols/sugar acids*									
184	2.011	179.0561	2.29	C_6_H_11_O_6_^−^	Myo-inositol	111	+	+	+
185	2.964	125.0244	4.9	C_6_H_5_O_3_^−^	Maltol	79	-	+	+

Fl: flower, St: stem, L: leaf, * Indicates the [M + HCOOH]^−^; PA: glycero-phosphatidic acid; PC: phosphatidylcholine; PE: phosphatidyl ethanolamine; PI: phosphatidylinositol; PG: phosphatidylglycerol; MGMG: Mono galactosyl monoacylglycerol; DGMG: Di galactosyl monoacylglycerol (*n* = 3).

#### Characterization of phenolic acids

Phenolic acids are a diverse class of plant secondary metabolites with benzoic or cinnamic acid skeleton and present mainly as free or conjugated forms. Structurally, the presence of phenolic hydroxyl or methoxy groups promote the antioxidant activity mediated *via* breaking the chain reactions. As reported by^28^ hydroxycinnamic acid exhibited better antioxidant activity than hydroxybenzoic acid. A total of 23 phenolic acids were annotated in all *Nymphaea* organs as indicated in Table 1. Gallic acid, caffeic and ferulic acids represented the major forms detected mostly as glycosides, showing loss of sugar moiety to yield the phenolic acid aglycone^3^.

In the negative Electrospray ionization, the characteristic fragment ion at *m/z* 169 of gallic acid was detected in peaks 1–5,7,9, 11,15,19 and 21 with a product ion at *m/z* 125 due to further elimination of CO_2_ unit, Table 1, Suppl. Fig. S4^21^. In details, series of galloyl glycosides have been unveiled based on their MS/MS pattern. Peaks 3 and 4 displayed [M- H]^-^ at *m/z* (483.078, C_20_H_19_O_14_^−^) and (331.0671, C_13_H_15_O_10_^−^) for di-galloyl-*O*- hexose and galloyl-*O*-hexose respectively with a mass difference of 152 amu indicating the presence of an extra galloyl moiety, Suppl. Fig. S4B, C^12^. Moreover, glycosides of protocatechuic, vanilic and ferulic acids were annotated in peaks 6,10,17 based on their corresponding aglycones at *m/z* at 153, 167 and 193, respectively^12^. Peak 16 was previously identified in Bangladesh *N. nouchali* stem and annotated as brevifolincarboxylic acid [M-H]^-^ at *m/z* (291.0146, C_13_H_7_O_8_^−^) based on characteristic fragment ions at *m/z* 247.02 and 219, and 191.04 due to losses of CO_2_ and CO moieties, respectively^12^.

In line with^12^ who reported several phenolic acids have demonstrated a good antioxidant potential radical. Specifically, gallic acid significantly attenuated the cellular oxidative stress induced by t-BHP (Tert-Butyl hydroperoxide). Similarly, protocatechuic and vanillic acids augmented the endogenous antioxidant defense system and provided cellular protection against oxidative stress.

#### Characterization of tannins

Ellagic acid derivatives constituted the major form of tannins in *Nymphaea* classified into two groups: galloyl hexoside and ellagitannins (ETs), in which two different derivatives of gallic acid are typically linked to hexahydroxy diphenic acid (HHDP) acid. Interestingly, galloyl hexoses represent the main precursors for ETs. Ellagitannins exhibited high structural diversity resulting from the variations in frequency, position of the HHDP unit, number of the galloyl moiety^29^.

Several structures derived from ellagic acid or dilactone form of HHDP were detected in peaks 24–49 Suppl. Fig S5. They were annotated based on their characteristic losses of 302 and 332 amu corresponding to HHDP and galloyl-*O*-hexose respectively. Likewise, peaks 27 and 39 [M-H]^−^ at *m/z* (633.0733, C_27_H_21_O_18_^−^) and (935.0796, C_41_H_27_O_26_^−^) showed a diagnostic loss of 302 amu ( M-H- HHDP) to yield 300.9982 in peak 27, while peak 39 showed extra loss of 332 amu corresponding to a galloyl hexoside moiety, Suppl. Fig. S5B, C^3^. Peaks 27 and 29 were annotated as corilagin and casuarictin, respectively, and in accordance with^30^ who reported them in *N. alba* L. leaves.

Geraniin peak 34 is a typical water-soluble ellagitannin with a potential antioxidant capacity, which yields upon complete hydrolysis of gallic acid and ellagic acid. Whereas, corilagin and brevifolin carboxylic acids were produced upon its partial hydrolysis at high temperature^3,31^. Importantly, geraniin and its hydrolysate gallic acid and corilagin showed substantial inhibitory activities against the aggregation of A*β* peptides, the main toxic components of senile plaques that play a crucial role in AD progression^32^. ETs were detected as major secondary metabolites in Egyptian leaves of *N. alba* L., and in accordance with^30^.

#### Characterization of flavonols

Flavonols are the most ubiquitous class of the flavonoid family with 3-hydroxyflavone backbone. Quercetin, patuletin, kaempferol, myricetin, and isorhamentin along with their conjugates were the most abundant flavonol identified in *Nymphaea* organs, Table 1, Suppl. Fig. S6. Structurally, the typical fragmentation pattern of *O*-glycosides showed neutral losses of sugar moieties that is 132 amu for pentose, 146 amu for rhamnose, 162 amu for hexose, and 308 for rhamno-hexosyl moieties yielding finally their corresponding deprotonated aglycone[Y^0^]^−^, and the radical aglycone [Y^0^-H] ^−^^22^ .In addition, losses of 152 amu and 42 amu^21^corresponding to galloyl and acetyl groups, respectively. This study represents the first report for patuletin glycosides occurrence in Egyptian *Nymphaea.*

A total of 46 flavonols glycosides were detected (Table 1), mostly as monoglycosides, acylated monoglycosides and di-glycosides. In detail, patuletin the rare flavonol and its derivatives were annotated in peaks 62, 66, 73, 76, & 84. In details, peaks 62 and 66 exhibited [M-H]^+^ at *m/z* (647.1243, C_29_H_27_O_17_^+^),and ( 495.1133, C_22_H_23_O_13_^+^) with elimination of 314 (152 + 162amu ) for galloyl hexosyl, and 162 amu for hexose moieties respectively, and ultimately yielding patuletin aglycone at *m/z* 333, annotated as patuletin-O-galloyl hexoside, and patuletin-*O*-hexoside respectively, Suppl. Fig. S7. Interestingly, patuletin exhibited multiple biological activities viz.; cytotoxic, antimicrobial, anti-inflammatory and neuroprotective^33^. Importantly, where patuletin glycosides were not previously identified in fully blooming *N. rubra* from Bangladesh^3^.

Similarly, peaks 64 and 88 displayed [M-H]^−^ at *m/z* (615.0992, C_28_H_23_O_16_^−^) and (473.1089, C_23_H_21_O_11_^−^) with a characteristic loss of galloyl hexose (152 + 162amu) and acetyl rhamnoside (146 + 42) moieties with generation of their aglycone at 301 and 284 respectively^34^. Therefore, peaks 64and 88 were annotated as quercetin-*O-*galloyl hexoside and kaempferol-*O*-acetyl-rhamnoside, Suppl. Fig. S8. respectively. In line with these results, kaempferol-7-*O*-galloylgalactosyl-rhamnoside and quercetin-3-*O*-acetylgalactoside were previously identified in the petals of Chinese water lily^35^. Specifically, acylated flavonoids exhibited higher bioavailability and penetration power attributed to their lipophilic nature^36^.

In an animal model, quercetin glycosides were significantly attenuated the inflammatory markers and inhibited the neuroinflammation in mice hippocampus. Moreover, they could reverse lipopolysaccharide induced synaptic loss through reduction of caspase-3 activity in the cortical and hippocampal regions^37^.

Among other flavonoid, 13 myricetin glycosides and 7 isorhamnetin glycosides were identified mainly as acylated glycosides with gallic and coumaric acids. Structurally, in the MS/MS spectrum revealed the presence of the deprotonated aglycone fragments at *m/z* 317 and 314, as shown in Table 1, indicated that these compounds were originated from myricetin and isorhamnetin, respectively.

For instance, peaks 70 and 81 exhibited [M-H]^-^ at *m/z* (505.0969, C_23_H_21_O_13_^−^) and (477.1402, C_23_H_25_O_11_^−^) with simple cleavages of consecutive glycosidic bonds generating daughter ion peaks at *m/z* 316 [M-H-146-42]^−^ and 315 [M-H-162]^-^ due to losses of acetyl hexoside and hexoside moieties, respectively. Consequently, they were annotated as myricetin-*O*-acetyl rhamnoside and isorhamnetin-*O*-hexoside, Suppl. Fig. S9^38^.

Regarding the medicinal importance, myricetin glycosides exhibited a potent antioxidant activity and could be used as an alternative to ascorbic acid, albeit less efficiently. Importantly, myricetin itself has been approved by the FDA as an important ingredient in foods, and health products with an excellent safety margin^39^. Similarly, isorhamnetin isolated from *Oenanthe javanica* as the main constituent significantly reduced the A*β*-triggered secretion of interleukin (IL- 6) and nuclear factor-*κ*B (NF-*κ*B). Evidence suggested that the neuroprotective effect of isorhamnetin was mediated through suppression of excessive ROS production and inhibition of TLR4 (toll-like receptor 4)^40^.

#### Characterization of flavanones

Naringenin and eriodictyol glycosides are the unique citrus flavanone with a broad spectrum of biological activities viz.; antioxidant, anti-inflammatory, anticancer, and neuroprotective effects^41,42^. Indeed, peaks 100 and 103 displayed [M-H]^−^ at *m/z* (433.1129, C_21_H_21_O_10_^−^) and (449.1089, C_21_H_21_O_11_^−^) with a characteristic loss of hexose moiety to give their corresponding aglycones at 271 for naringenin and 287 for eriodictyol and annotated as naringenin-*O*-hexoside and eriodictyol-*O*-hexoside, respectively, Suppl. Fig. S10^43^.

Intesetingly, naringenin significantly improved the spatial learning in lipopolysaccharide induced cognitive impairments through reduction of tumor necrosis factor *α* (TNF*α*), the nuclear factor-kappaB, (NF-κB), toll-like receptor 4 (TLR4), and ameliorated the cholinergic function *via* inhibition AChE enzyme^41^. Similarlly, eriodictyol obviously alleviated the memory impairment by suppression of A*β* aggregation, Tau phosphorylation in HT-22 Mouse Hippocampal Neuronal Cell Lines as a representive model for early diagnosis of Alzheimer’s disease^42,43^. However, to the best of our knowledge this is the first report about their identification in Egyptian lily.

#### Characterization of anthocyanins

Anthocyanins are pH sensitive agronomical traits in many fruits and ornamental flowers. Importantly, they play a crucial role as visual signals to attract pollinators and serve as a natural source of food colourants^34^. Globally, there is an increasing demand for pigments of natural origin. Brilliant Blue (E133) and Blue V (E131) were the only approved food additives by the US Food and Drug Administration. Unfortunately, several cases of hyperactivity, sleep disorders, and food intolerance were reported after consumption of jelly, soft drink, and candy colored with artificial dyes, especially in children. Natural red, orange, and yellow pigments are usually available, however, only a few sources are known as a natural source of blue colour^44^. Structurally, delphinidin glycosides were the predominant anthocyanins in *Nymphaea* flowers with a characteristic fragment ion of the parent aglycone at *m/z* 303 as observed in peak 108–113 .

Delphinidin is the primary anthocyanin responsible for the blue and purple colors of flowers. Glycosylation, hydroxylation, and acylation are an important step responsible for generation of the unique blue colour^44^.

This structural modification of anthocyanin significantly influences antioxidant activity. delphinidin glycoside derivative extracted from the blue pea flower clearly attenuated H_2_O_2_-induced cytotoxicity in human keratinocytes^44^.

#### Characterization of alkaloids

Spermidine alkaloids are ubiquitous class of organic polyamine where an aliphatic chain of triamine is connected to flexible methylene groups. They exhibit special chemophenetic significances as they stabilize the nucleic acid structure, protein synthesis, and cell proliferation and serve as a nitrogen reservoir for the fundamental cellular processes^45^. Unlike flavonoids, spermidine alkaloids were tentatively identified in ESI-MS in the positive mode and mainly concentrated in *N*. flowers Suppl. Fig. S11B. The MS/MS spectra showed that these alkaloids were typically substituted with cinnamic acid derivatives viz.; *p*-coumaric acid, ferulic acid and caffeic acid at N-1, N-5, or N-10 to produce spermidine conjugate^45^. Indeed, they showed an intense ion at [M + H]^+^ at *m/z* 147 corresponding to spermidine. Peak 117 showed [M + H]^+^ at *m/z* (468.2493, C_26_H_34_N_3_O_5_^+^) with an intial loss of coumaroyl moiety (146amu) followed by a neutral losses of feruloyl group (176amu) to yield fragment ions at *m/z* 322 and 147 respectively, and assigned as feruloyl coumaroyl spermidine Suppl. Fig S12A. Similarly, cleavages of amide bonds of trisubstituted-amine were observed in peak 118 (584.2755, C_34_H_38_N_3_O_6_^+^) with a successive losses of three coumaroyl moeities to produce 438, 292, 147 respectively, and identified as tricoumaroyl spermidine Suppl. Fig S12B. Both spermidine alkaloids were previously detected in Japanese tea flowers^46^. In agreement with^8^, both apomorphine and nuciferine were virtually absent in the blue lotus extract.

Recently^45^ documented AChE inhibitory activities of spermidine alkaloids of *Lycium barbarism* in transgenic Alzheimer drosophila model. More importantly^45^ outlined the essential role of spermidine as a natural autophagy inducer to eliminate the aggregates of misfolded brain proteins that involved in the pathological changes of AD. To the best of our knowledge, this is the first report about the presence of spermidine alkaloids in Egyptian lily.

#### Characterization of amino acids and vitamins

Vitamins are a group of structurally diverse group of micronutrients that play an essential role as coenzymes in the critical cellular functions while amino acids are the main building block of protein. Despite the extensive research on nutritive value of *Nymphaea*, however, the available data about vitamins and amino acids contents was scarce. Several vitamins and amino acids were tentatively identified in the positive mode. Among the most important vitamin B-group, pantothenic acid (vitamin B-5) is an important obligate precursor of brain acetyl-CoA which is necessary for synthesis of the neurotransmitter acetylcholine. It was able to reverse the causative agents in in sporadic Alzheimer’s disease^47^. Nicotinamide is another water soluble vitamins of group-B family. Recently, in metabolomics based translational study nicotinamide was identified as a potential biomarker for Alzheimer’s disease^48^. Peak 129 exhibited [M + H]^+^ at *m/z* (161.1073,C_10_H_13_N_2_^+^) with intial elimination of the terminal amino group(M + H-17) to give a fragment ion at *m/z* 144 followed by an intense ion at *m/z* 80 Suppl. Fig S13^49^.

#### Characterization of organic acids

A total of 7 organic acids, were tentatively identified in the negative mode where malic, citric acid, succinic acid and shikimic acid were the most abundant acids Table 1. Malic acid is one of the most ubiquitous dicarboxylic acid that is widely used to improve flavor in the food and beverage industries. Currently, it is approved by the U.S. Food and Drug Administration (FDA) as safe food-grade additive^50^. Malic acid peak 130 was identified as the unique compound in the blue flowers with [M + H]^-^ at *m/z* (133.0142, C_4_H_5_O_5_^−^) with a diagnostic loss of water molecule to produce a fragmentation ion at *m/z* 115 followed by decarboxylation to yield an intense ion at *m/z* 71,(Suppl. Fig S14A). A similar fragmentation pattern was observed with citric acid, peak 132 at *m/z* (191.0197, C_6_H_7_O_7_^−^) and succinic acid at *m/z* (117.0193, C_4_H_5_O_4_^−^), Suppl. Fig S14 B, C^21^.

#### Characterization of fatty acids & amides

A total of 13 fatty acids (FAs) along with their hydroxylated and amides derivatives were putatively detected. Structurally, linolenic, oleic, and palmitic acids were almost uniformly distributed in all lily organs. Fatty acids are an important source of energy, mainly stored in adenosine triphosphate(ATP) the universal energy source for cells^24^. Whereas, fatty acid amides (FAAs) are essential bioactive signaling molecules with several pharmacological activities viz.; analgesic, anti-anxiety, anti-convulsion, and neuroprotective effects^54^.

The typical fragmentation pattern of fatty acids started with a neutral loss of water^51^ followed by decarboxylation as illustrated in peak 145 assigned as hydroxy-palmitic acid [M-H]^−^ at *m/z* (271.2279, C_16_H_31_O_3_^−^), Suppl. Fig S15A. Peak 147, [M + H]^+^ at *m/z*(282.2791, C_18_H_36_NO^+^) was assigmed as oleamide due to successive elimination of NH_2_ and water molecule to yield 265, 247 respectively, Suppl. Fig S15B^52^.

#### Characterization of lipids

Lipids are typically consist of a glycerol backbone attached to two fatty acid tails and a phosphate group. They are the most abundant insoluble biomolecules of the cell membrane and can be divided into several classes viz.; sphingolipids, sulfolipids, glycerolipids, and phospholipids based on the nature of polar head that linked to glycerol backbone. The structural diversity of phospholipids depends on FAs acyl chains that linked at the sn-1 and sn-2 positions with phosphate group along with the glycerol backbone. They play a crucial role in cell protection and act as a barrier against environmental insults^53^.

**Fig. 2:**
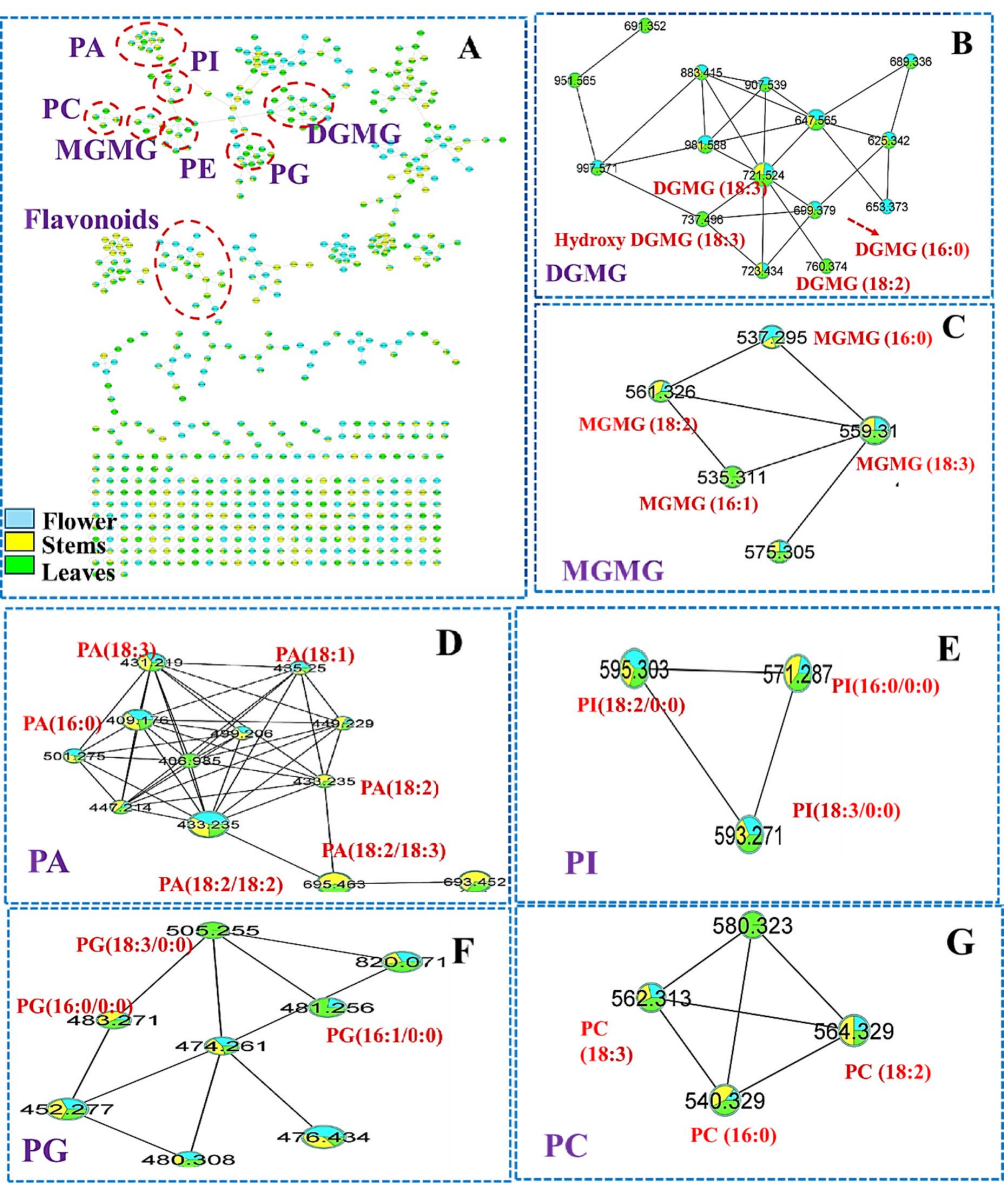
(**A**) Molecular network of *Nymphaea nouchali* extracts; flower, stem and leaves based on MS/MS in negative mode. Selected nodes and clusters have been zoomed in viz.; (**B**) DGMG (Di-galactosyl monoacylglycerol); (**C**) MGMG (Mono galactosyl monoacylglycerol); (**D**) PA (glycero-phosphatidicacid); (**E**) PI(phosphatidylinositol); (**F**) PG (phosphatidylglycerol); (**G**) PC(phosphatidylcholine).

Several lipids (33) were tentatively identified in all *Nymphaea* organs belonging to different classes viz.; glycero-phosphatidic acid (PA), phosphatidylcholine (PC), phosphatidyl ethanolamine(PE), phosphatidylinosito (PI), phosphatidylglycerol (PG), Mono galactosyl monoacylglycerol (MGMG) and Di galactosyl monoacylglycerol (DGMG ) as illustrated in Fig. 2. In detail, the MS/MS of peak 155 [M-H]^−^ at *m/z* (577.2688, C_27_H_45_O_11_S^-^) demonstrated an intense fragment ion of sulfoquinovosyl moiety at *m/z* 225 with a diagnostic fragment of sulfonate moiety at *m/z* 80. Accordingly, it was annotated as sulfoquinovosyl monoacyl glycerol (18:3) Suppl. Fig S16A^24^. Regarding the phosphatidyl ethanolamine (PE),4 compounds were assigned in peaks 157, 164, 167, and 177. For example peak 157 [M-H]^-^ at *m/z* (474.2610, C_23_H_41_NO_7_P^-^) which showed a characteristic product ions at *m/z* 277 and 196 indicating the presence of linolenic and phosphatidyl-ethanolamine moeities respectively, and annotatted as PE (18:3/0:0) Suppl. Fig. S16B^57^.

**Fig. 3:**
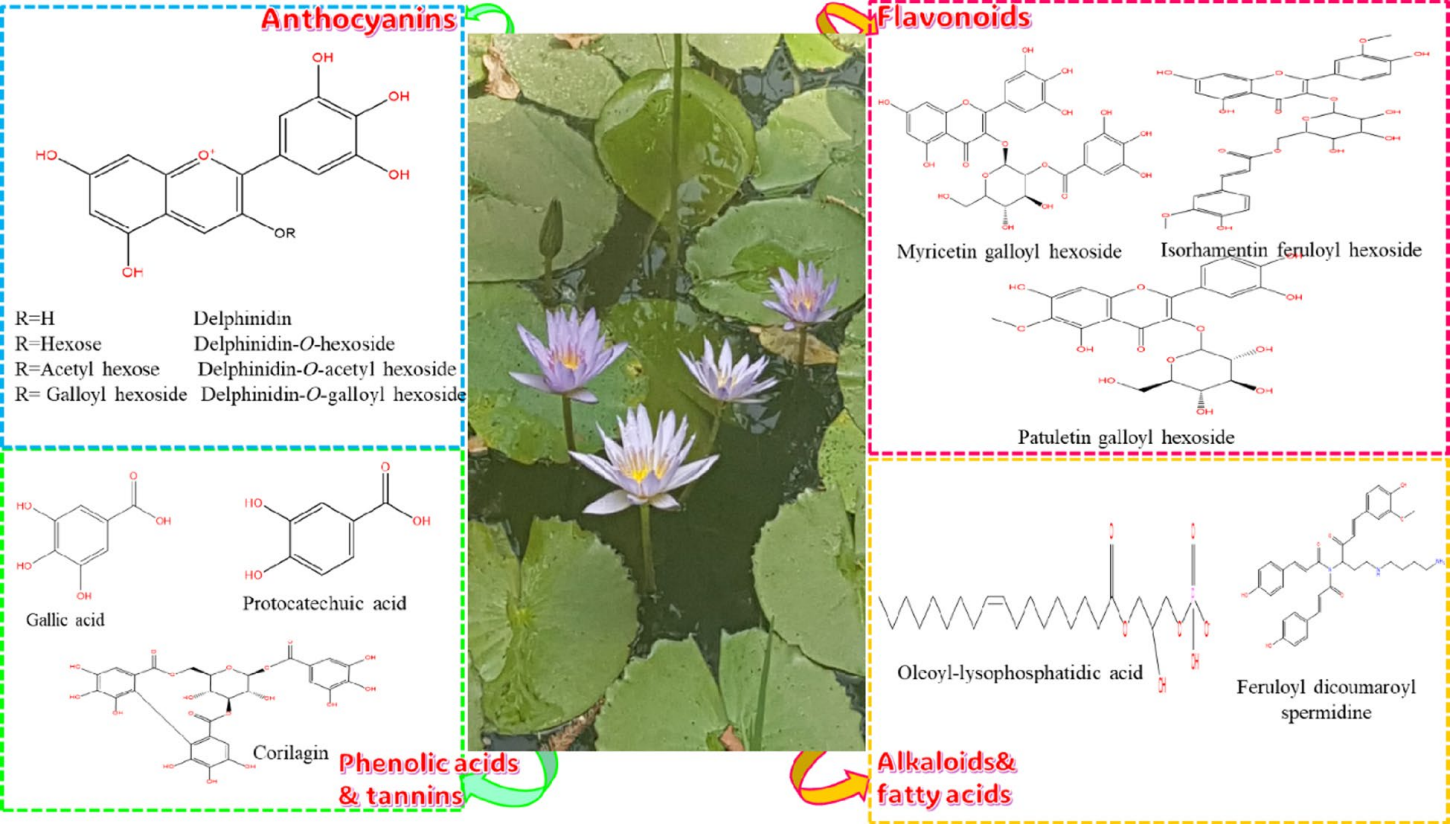
Structure of selected secondary metabolites tentatively identified in *Nymphaea nouchali* var. caerulea organs.

Interestingly, 4 mono galactosyl monoacylglycerol (MGMG )and 4 di-galactosyl monoacylglycerol (DGMG) were identified as negative mode as formate adducts [M + CH_3_COO]^−^ ions Table 1; Fig. 3B,C. The fragmentation was intiated by a diagnostic loss methyl formate (60 amu) to libarate their corresponding fatty acid 277 for octadecatrienoic as in peak 163 (559.3110, C_27_H_45_O_9_^−^ ), Suppl. Figure 16B and assigned as MGMG (18:3)^54^. Recently, MGMG has attracted a special interest attributed to its multiple biological effects as antioxidant, anti-hyperlipidemic and anti-inflammatory activities^55^.

Similarly, glycero-phosphatidic acid (PA) were annotated in peaks,151, 161,172, 173,176, 180, and 181 Fig. 2D. Peak 172 [M-H]^−^ at *m/z* (433.2350, C_21_H_38_O_7_P^−^) was assigned as PA(18:2)based on the diagnostic loss of linoleic acid to yield a fragment ion at *m/z* 153 (phospho-glycerol) and phosphonate group at *m/z* 79, Suppl. Fig. S17B^57^. Importantly, PA is the primary building block of in the synthesis of phospholipid and its unique geometry can alter the membrane topology. Moreover, PA acts as a functional messenger in intracellular signaling pathways and it defeciency is directly linked to the progession progression of AD^56^. Moreover, phosphatidylinositol (PI), Fig. 2E was tentatively identified based on the presence of several characteristic ions at *m/z* 241(cyclic anion of inositol phosphate), 153 (phospho-glycerol)^24^.

For the best of our knowledge, this is the first comprehensive metabolite profiling of 33 lipids in *Nymphaea* and the structures of selected secondary metabolites tentatively identified in *N. nouchali* var. caerulea organs were illustrated in Fig. 3.

## Multivariate data analysis (MVA) of N. nouchali organs

Owing to the intricacy of the obtained data of three replicates (*n* = 3) of *N. nouchali* organs specimens (flower, leaf and stem) and monitored secondary metabolites Table 1, MVA was further employed in an untargeted manner including principal component analysis (PCA), hierarchical cluster analysis (HCA), and orthogonal partial least square (OPLS) analysis for specimens classifcation in an untargeted manner. To better designate similarities and differences among organs as a trial to guarantee better analytical rigorousness.

### HCA and PCA analyses of ESI–QTOF–MS/MS extracted secondary metabolites

**Fig. 4:**
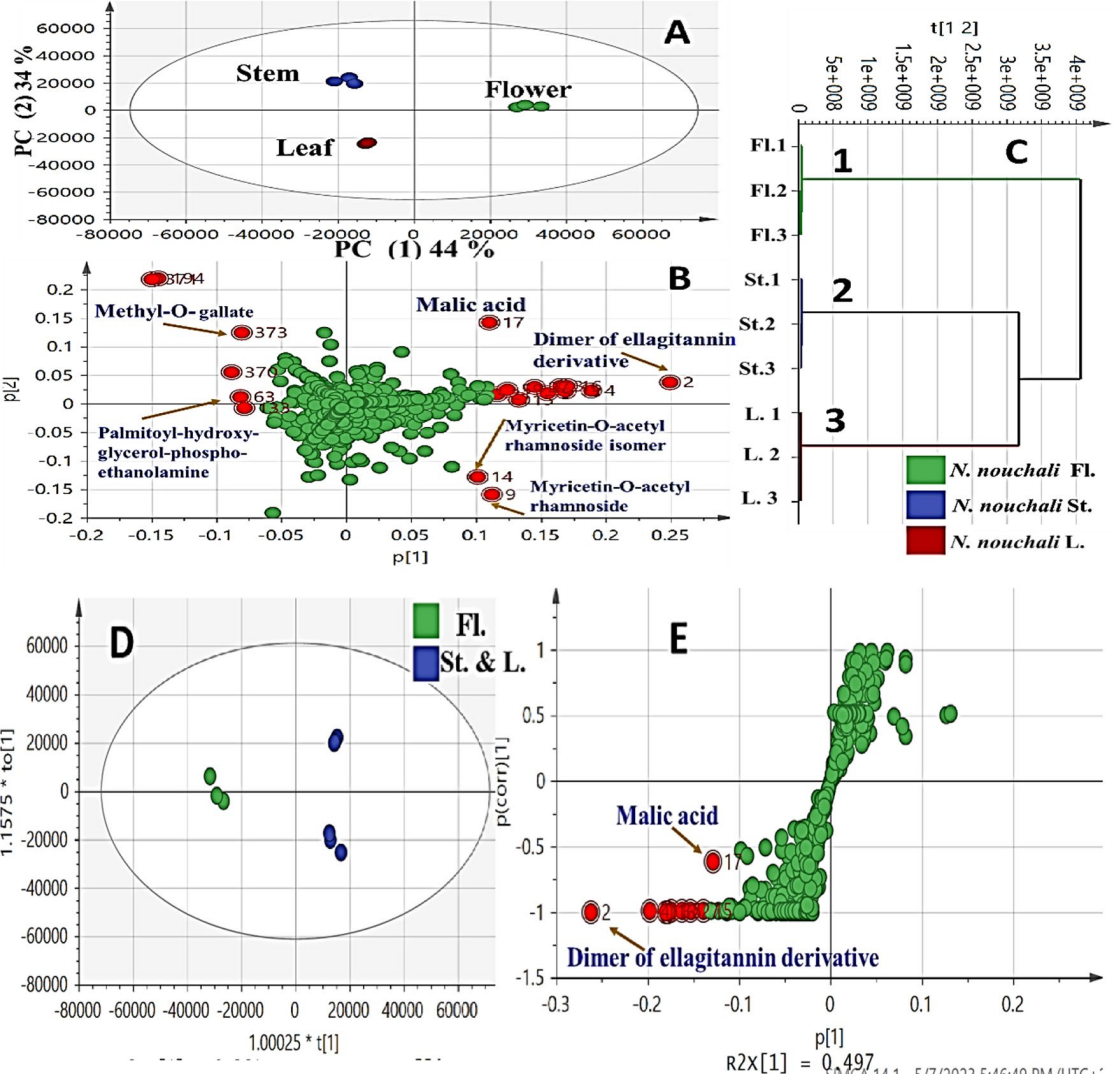
UPLC-based principal analyses of *N. nouchali* organs viz. flower, stem and leaf in negative mode (*n* = 3). The metabolome clusters are located at the distinct positions described by two vectors of PC1 (44%) and PC2 (34%) with constituent denoted by its base peak mass value. (**A**) Score plot of PC1 versus PC2 scores. (**B**) Loading plot for PC1 and PC2. (**C**) HCA dendrogram analysis. (**D**) OPLS-DA score plot and (**E**) loading S-plots. The S-plot shows the covariance p[1] against the correlation p(cor)[1] of the variables of the discriminating component of the OPLS-DA model. Cut-off values of *P* < 0.01 were used.

PCA and HCA were internally performed for all specimens viz.; flower, stem and leaf. In negative ion mode, PCA score plot Fig. 4A showed PC1 versus PC2 with total variance of (78%) where PC1 (44%) and PC2 (34%). PCA loading plot Fig. 4A explains the discrimination of samples in terms of secondary metabolites.

Within the right side of Fig. 4B that corresponds to cluster (1) Fig. 4C which included flower specimens, found clearly segregated due to presence of ellagitannin derivative and myricetin-*O*-acetyl glycoside isomers followed by malic acid Table 1; Fig. 4B suggesting their potential as a marker for flower of Egyptian *N. nouchali*. On the other side, both stem and leaf were clustered together being enriched in palmitoyl-hydroxy-glycerol-phospho-ethanolamine and methyl-*O*- gallate as shown in parts Fig. 4B,C. Higher variance coverage was observed in the negative ion mode derived PCA model. Such model showed an obvious discrimination of the flower at the right side of PC1 as shown in Fig. 4B.

**Fig. 5:**
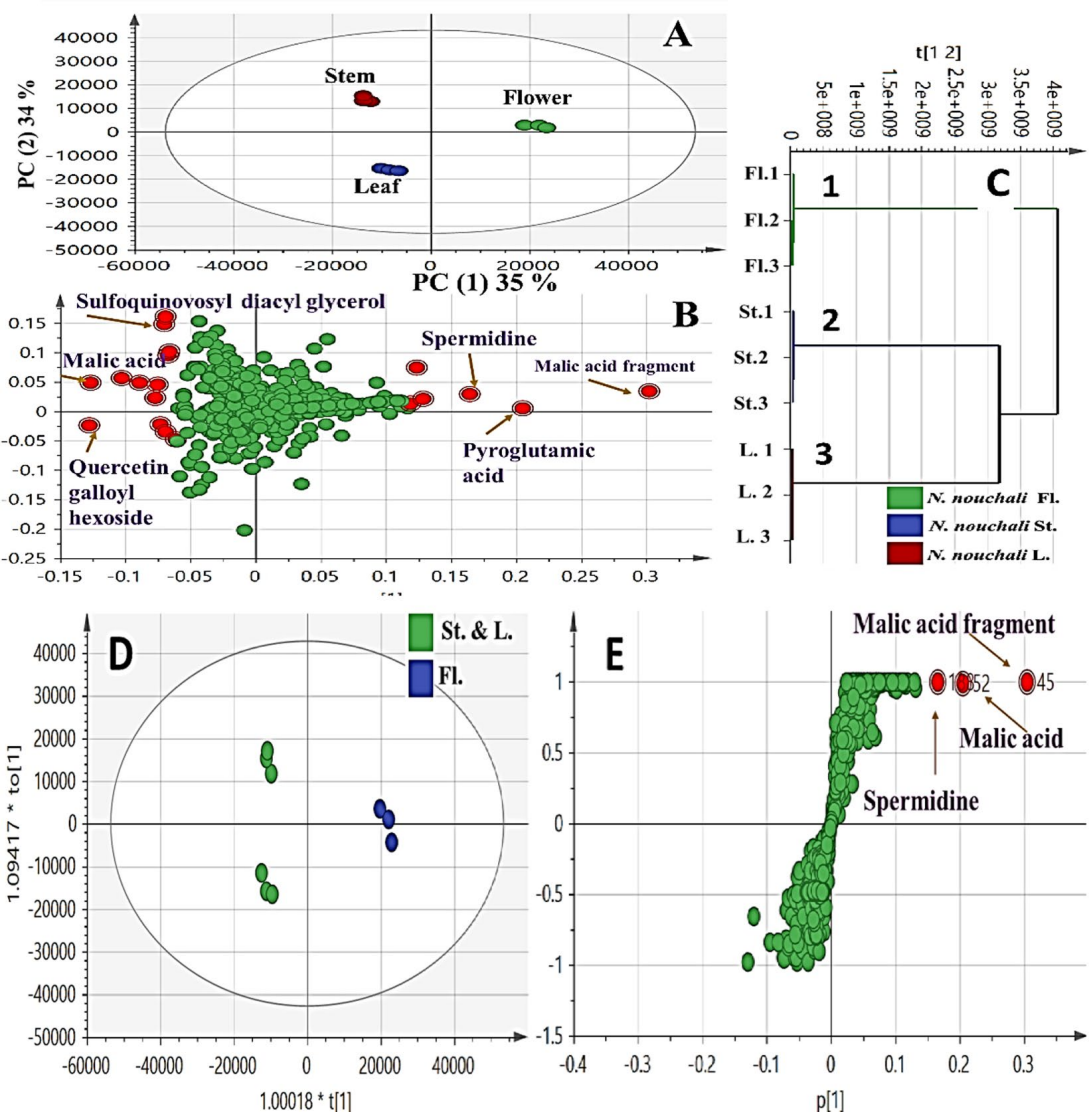
UPLC-based principal analyses of *N. nouchali* organs viz. flower, stem and leaf in positive mode (*n* = 3). The metabolome clusters are located at the distinct positions described by two vectors of PC1 (35%) and PC2 (34%) with contributing mass peaks and their assignments. (**A**) Score plot of PC1 versus PC2 scores. (**B**) Loading plot for PC1 and PC2. (**C**) HCA dendrogram analysis of *N. nouchali* organs based on group average cluster analysis. (**D**) OPLS-DA score plot and (**E**) loading S-plots. The S-plot shows the covariance p[1] against the correlation p(cor)[1] of the variables of the discriminating component of the OPLS-DA model. Cut-off values of *P* < 0.01 were used.

Meanwhile, the positive ionization mode showed PCA score plot Fig. 5A with PC1 (34%) versus PC2 (35%) with total variance of (69%). Samples’ segregation was attributed to malic acid, pyroglutamic acid and spermidine, on the right side enriched in flower. Spermidine exerts neuroprotective effect against degenerative changes that is mediated through its antioxidant and anti-inflammatory properties^57^. Meanwhile, quercetin galloyl hexoside and malic acid followed by sulfoquinovosyl diacyl glycerol showed the segregation of both stem and leaf on the right side of the loading plot Table 1; Fig. 5B for PC1 and PC2 of positive ionization mode.

(HCA) dendrogram, Fig. 4C of the negative mode displayed the clustering pattern of samples, displayed two distinct clusters viz.; cluster (1) included the flower of Egyptian *N. nouchali*, that was segregated from cluster (2) and (3) that comprised stem and leaf samples. The same result was concluded from (HCA) dendrogram Fig. 5C laid on positive mode ionization.

The detection of alternative markers in both negative and positive ionization modes for flower indicated the importance of acquiring different ionization modes to provide a broader metabolome coverage. This was detected by a higher segregation pattern with a total covered variance of 72% in negative mode versus 69% in positive mode.

### OPLS of flower extracted secondary metabolites from stem and leaf

Supervised orthogonal projection to latent structures-discriminant analysis (OPLS-DA) was utilized to confirm the revealed markers. OPLS-DA exhibited high value in the identification of markers consequently, samples classification, and by providing the most relevant variables^23^.

Examination of the OPLS score plot in negative mode Fig. 4D clearly separated flower at the right side from others (leaf and stem) at the left one due to prevalence of methyl-*O*- gallate and palmitoyl-hydroxy-glycerol-phospho-ethanolamine in the later. Such separation of flower was ascribed of its richness in ellagitannin derivative as revealed from the loading plot Fig. 4E. Malic acid were predominating in flower, and constituted the main anti-inflammatory effect.

The loading plot derived from secondary metabolites dataset OPLS Fig. 4D, E in negative mode ionization revealed the most relevant variables assigned (R^2^ = 0.99) with a prediction goodness parameter Q^2^ = 0.97.

### OPLS- DA analysis of flower versus stem and leaf

Flower specimens were modelled against others viz., stem and leaf specimens, as one class group, with the derived score plot showing a considerable separation between both categories Fig. 5D, E. The OPLS score plot gained 78% of the total variance with the prediction goodness parameter Q2 = 0.69 and R2 = 0.57 Fig. 5D. In OPLS-DA, a particularly useful tool that produced variable importance in projection (VIP) scores for each metabolite. The validation model was achieved using the diagnostic metrics Q2, R2, permutation testing, and *P*-value to avoid overfitting and assess the statistical significance of the model.

As revealed from OPLS-DA, the characteristic secondary metabolites that have the potential to discriminate flower Fig. 5E from others included: malic acid and spermidine as characteristic markers for discrimination of flower, and in agreement with PCA results, Fig. 5B.

Partial least squares (PLS) analysis can be used for deriving a correlation between the bioactivities and the identified phytochemicals using LC-MS for identifying the main contributing variables as reported in our previous work: https://www.sciencedirect.com/science/article/pii/S0308814623004831. Unfortunately, this would need many fractions to allow a strong model be generated to predict bioactivity, which is not attained in our case as one extract was tested for bioactivity.

## Biological activities

### Determination of antioxidant activity

**Fig. 6:**
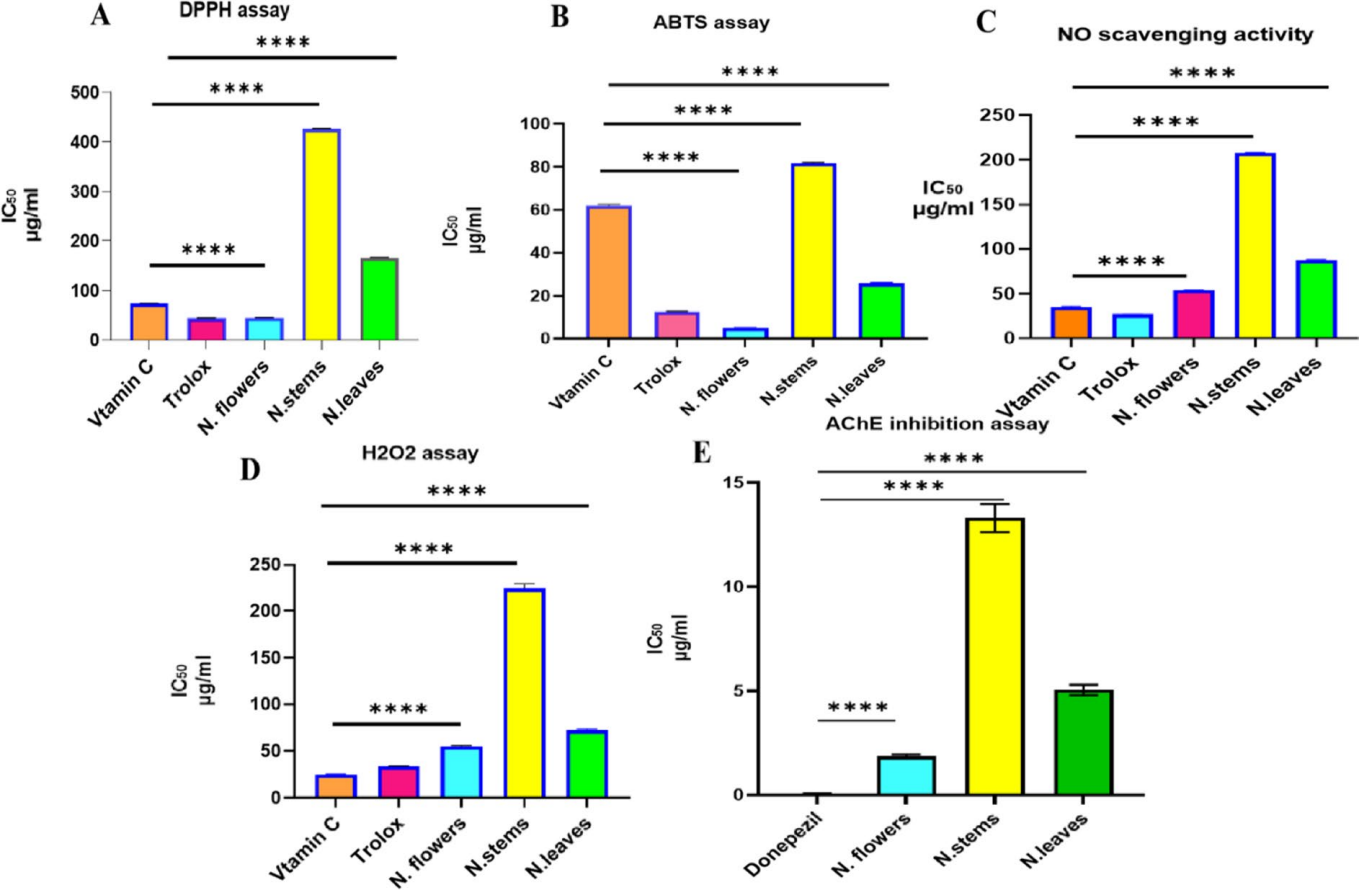
Antioxidant effects of N. *nouchali* extracts; flower, stems and leaves as IC_50_ value compared to reference materials; Vit C and Trolox. (**A**) DPPH scavenging activity, (**B**) ABTS^+^ scavenging activity, (**C**) NO scavenging activity, (**D**) H_2_O_2_ scavenging activity, and E: AChE inhibition activity.

For the assessment of the potential antioxidant activity, more than one assay was tested. All *Nymphaea* extracts exhibited a potential radical scavenging activity in 5 different assays viz.; DPPH, ABTS, NO and H_2_O_2_ scavenging. ABTS and DPPH assays are the most popular assays used for evaluation of the antioxidant potential based on the principle of single-electron transfer (SET) with a successive interruption of oxidative degradation chain^23^. Based on IC_50_ values, flowers recorded the highest DPPH scavenging capacity (45.15 µg/ml) as compared to IC_50_ of Trolox (43.96 µg/ml) and lower than Vit C (74.1 µg/ml), followed by leaves and stems, Fig. 6. Similarly, flowers recorded the highest ABTS^+^, with the lowest IC_50_ as compared to Vit C and Trolox, Fig. 6.

Hydrogen peroxide (H_2_O_2_) and nitric oxide (NO) are essential redox signaling molecules. Under certain pathological conditions, H_2_O_2_ generates highly reactive hydroxyl radicals that lead to widespread perturbation of many cellular and organelle membranes. Similarly, excessive production of NO can trigger a successive tissue damage^58,59^.

Interestingly, flavonoids as isorhamnetin the primary bioactive metatolite significantly suppressed the secretion of the pro-inflammatory mediators TNF α, interleukin (IL) 1β, and IL-6 tar geting nuclear factor (NF)-κB and Toll-like receptor 4(TLR) in monocyte/macrophage-like cells^60–62^consequenly, quell the oxidative stress.

As illustrated in Fig. 6, flowers exhibited pronounced scavenging activity, with IC_50_ of 55.04 and 54.07 µg/ml in H_2_O_2_ and NO assays, respectively.

### Anticholinesterase activity

As illustrated in Fig. 6E, flowers demonstrated the best AChE inhibition activity. Meanwhile, stem showed the least activity compared to donepezil standard AChE activity. Currently, donepezil is one of FDA approved AChE inhibitor drug. It only provides a symptomatic relieve of the symptoms but does not stop the progression of Alzheimer’s disease with several cholinergic side effects. Unlike donepezil, natural plant based metabolites have been explored as a safe alternative approach to reduce alzheimer’s symptoms. Recently, they have gained significant attention due to their unique effects and multi-targets^41^.

As illustrated in Fig. 6E, flowers demonstrated the best AChE inhibition activity. Meanwhile, stem showed the least activity compared to doneperzil standard AChE activity. In line with^61^ who revealed the tentative identification of fifty-three compounds in *N.alba* leaf by UPLC-MS/MS analysis with antioxidant and anti-inflammatory activities. In a good agreement with^3^, ethyl acetate of *Nymphaea rubra* flowers exhibited a potent antioxidant, α-glucosidase, and elastase activities.

Similarly^62^ highlighted that ethyl acetate extract of *N. candida* Presl showed significant antidepressant effects mediated through reduction of neuroinflammation and mitigation of oxidative stress.

Interestingly, delphenidin was the most abundant anthocyanin in flower extract with a broad spectrum of health benefits viz.: anticancer, anti-inflammatory, cardioprotective and antihypertensive activities. Importantly, its neuroprotective potential was mediated through inhibition of H_2_O_2_ induced oxidative stress and supression of A*β* amyloid beta induced glycogen synthase kinase associated in Alzheimer’s disease^60^.

Additionally, naringenin as the unique flower lipophilic flavanone exhibits a potent neuroprotective activity with high permeability to cross the blood–brain barrier. In an animal model, it can upregulate insulin signaling and improve the cognitive decline usually associated with Alzheimer disease^41^. More importantly, daily intake of spermidine alkaloids for 3 months improved the memory performance in aged people and suppressed the release of pro-inflammatory cytokines and accumulation of amyloid beta (A*β*)^61^.

There is a scarcity of research on the impact of use of chromatographic analysis to identify, characterize and compare bioactive constituents among organs of water lilies. Additionally, the authors examined the potential role of Egyptian *N. nucifera* extracts in mitigating the detrimental effects of oxidative stress and neurodegenerative disorders.

## Conclusion

Water lily has been recognized as an indigenous food item and plays a vital role as a nutraceutical. The results of our study provide a novel insight into the metabolome of different organs of Egyptian *N. nouchali*., viz., flower, stem and leaf. In this context, multivariate data analyses aided in organ classification alongside the identification of main biomarkers of each organ. The results provide a comprehensive metabolite profiling of secondary metabolites in blue lotus different organs *via* UHPLC/PDA/ESI-QTOF-MS coupled with molecular networking. A total of 185 metabolites were annotated belonging to phenolic acids (23), ellagitannins (26) flavonoids (58), anthocyanins (7), alkaloids (6), amino acids and vitamins (9), organic acids (7), fatty acids & amides (13), lipids (33) and sugars/sugar derivatives (2). Seventy-two compounds were reported for the first time in *N. nouchali*, especially phenolic acids as gallic acid and its derivatives, ellagitannins viz., ellagic acid & corilagin, and flavonoids as patuletin glycosides & naringenin eriodictyol glycosides, and lipids, and all adding to the complex chemical makeup of that taxa.

MN allowed for effective structural mapping and clustering of metabolites, while MVA highlighted compositional similarities and variations among the secondary metabolites investigated. Flower specimens were modelled against others viz., class group of stem and leaf specimens, and showed a considerable separation. OPLS-DA revealed characteristic markers for discrimination of flower, in agreement with PCA results. Moreover, flower extract was the richest in delphenidin anthocyanin, spermidine glcosides, and naringenin aglycone. *In-vitro* assays revealed the multi-target activities of water lily parts viz., antioxidant and anti-Alzheimer, especially for flowers with a superior effect compared to leaf and stem. Furthermore, it is essential to understand their metabolic pathways and neurological effects within the context of AD treatment and to conduct comprehensive well designed clinical studies to evaluate the therapeutic efficacy of these natural compounds.

## Supplementary Information

Below is the link to the electronic supplementary material.

Supplementary Material 1

## Data Availability

Data will be made available on request by emailing the corresponding authors at [inas.younis@pharma.cu.edu.eg](mailto: inas.younis@pharma.cu.edu.eg) .
