# Supplementary material for: Unravelling Egyptian blue Lily (Nymphaea nouchali) organs’ metabolome via UHPLC/PDA/ESI-QTOF-MS and in relation to their antioxidant and anti-cholinesterase effects
Source: Sci Rep. 2025 Dec 19;15:44136. doi: 10.1038/s41598-025-30937-y (PMC12717157; doi:10.1038/s41598-025-30937-y)
Supplement: Supplementary file 1 — Supplementary Material 1 [file 41598_2025_30937_MOESM1_ESM.docx]

**Supplementary Materials**

**Unravelling Egyptian blue lily (*Nymphaea nouchali*) organs’ metabolome via *UHPLC/PDA/ESI-QTOF-MS* and in relation to their antioxidant and anti-cholinesterase effects**

**Running Title:** Egyptian *Nymphaea caerulea metabolome and biological effects*

Inas Y. Younis ^a#^*****, Ahmed F. Essa ^b^ ^#^, Samah A. El-Newary ^c^, Abdelbaset M. Elgamal ^d^, Mohamed A. Farag ^a, e^, Engy Mohsen ^a,^*****

^a^ Pharmacognosy Department, Faculty of Pharmacy, Cairo University, Cairo, Egypt

^b^ Chemistry of Natural Compounds Department, National Research Centre, 33 El Bohouth St., Dokki, Giza 12622, Egypt.

^c^ Medicinal and Aromatic Plants Research Department, pharmaceutical industries research Division, National Research Centre, El-Bouhoths St., Dokki, Cairo 12622, Egypt.

^d^ Department of Chemistry of Microbial and Natural Products, National Research Centre, 33 El-Bohouth St., Dokki, Cairo 12622, Egypt^.^

^e^ Health care faculty, Saxony Egypt University (SEU), Badr City, Egypt

^#^ Both authors contributed equally

**Correspondence: Inas Y. Younis, Pharmacognosy Department, Faculty of Pharmacy, Cairo University, 11562, Cairo, Egypt.**

Email:  [inas.younis@pharma.cu.edu.eg](mailto:%20inas.younis@pharma.cu.edu.eg)

**C0-correspondence: Engy Mohsen, Pharmacognosy Department, Faculty of Pharmacy, Cairo University, 11562, Cairo, Egypt.**

Email:  [engy.mohsen@pharma.cu.edu.eg](mailto:%20engy.mohsen@pharma.cu.edu.eg)

**Suppl. Fig. S1** phenolic, flavonoids and tannins of *Nymphaea nouchali* extracts; flower, stem and leaves.


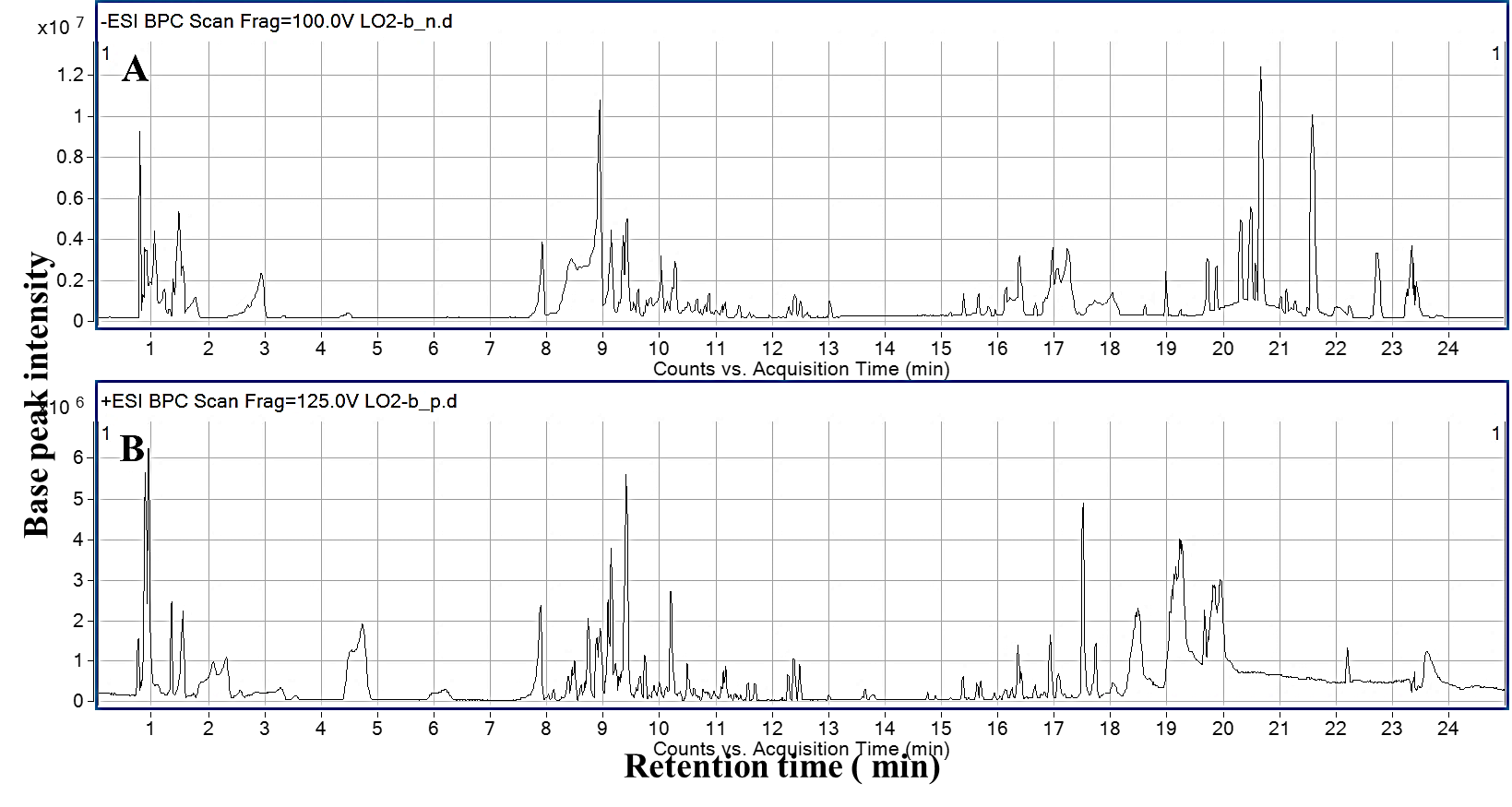


**Suppl. Fig. S2**. UPLC-q-tof-MS base peak chromatogram of *Nymphaea caerulea stems* ethanolic extract detected in (A) negative and (B) positive ionization modes.


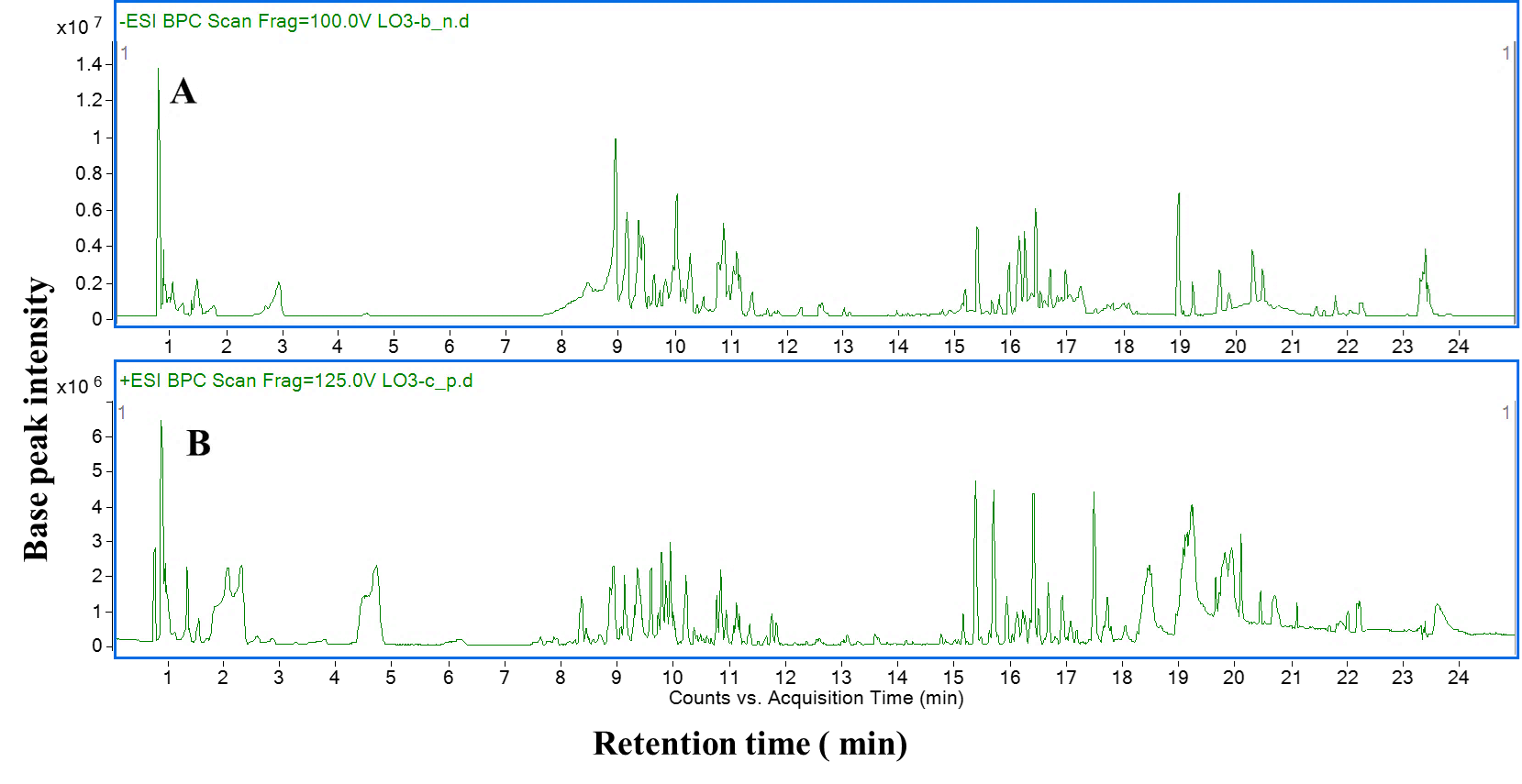


**Suppl. Fig. S3**. UPLC-q-tof-MS base peak chromatogram of *Nymphaea caerulea leaves* ethanolic extract detected in (A) negative and (B) positive ionization modes.


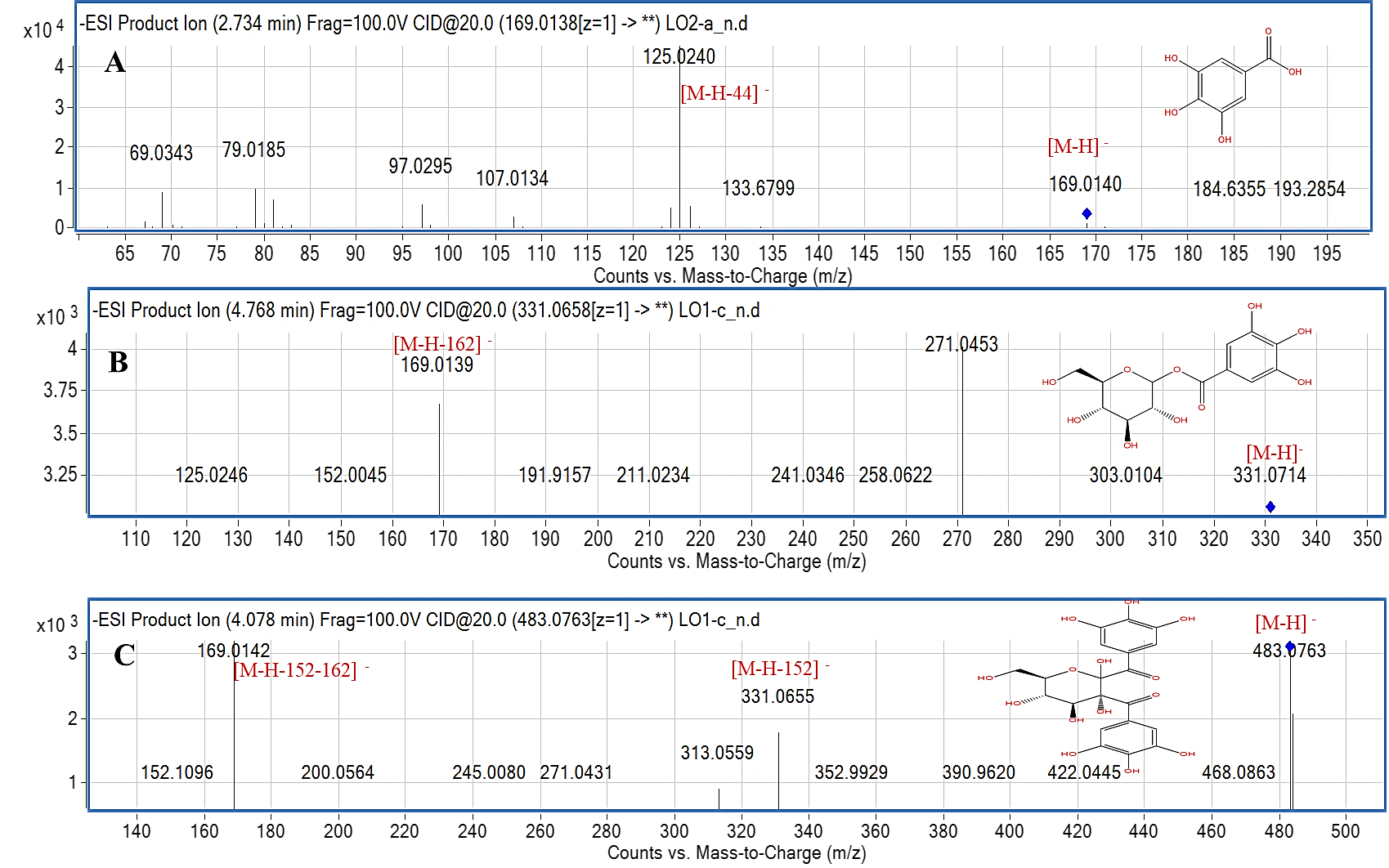


**Suppl. Fig. S4**. MS/MS spectra of (A) gallic acid, (B)galloyl-*O*- hexose, and (C)di-galloyl-*O*- hexose in the negative ion mode.


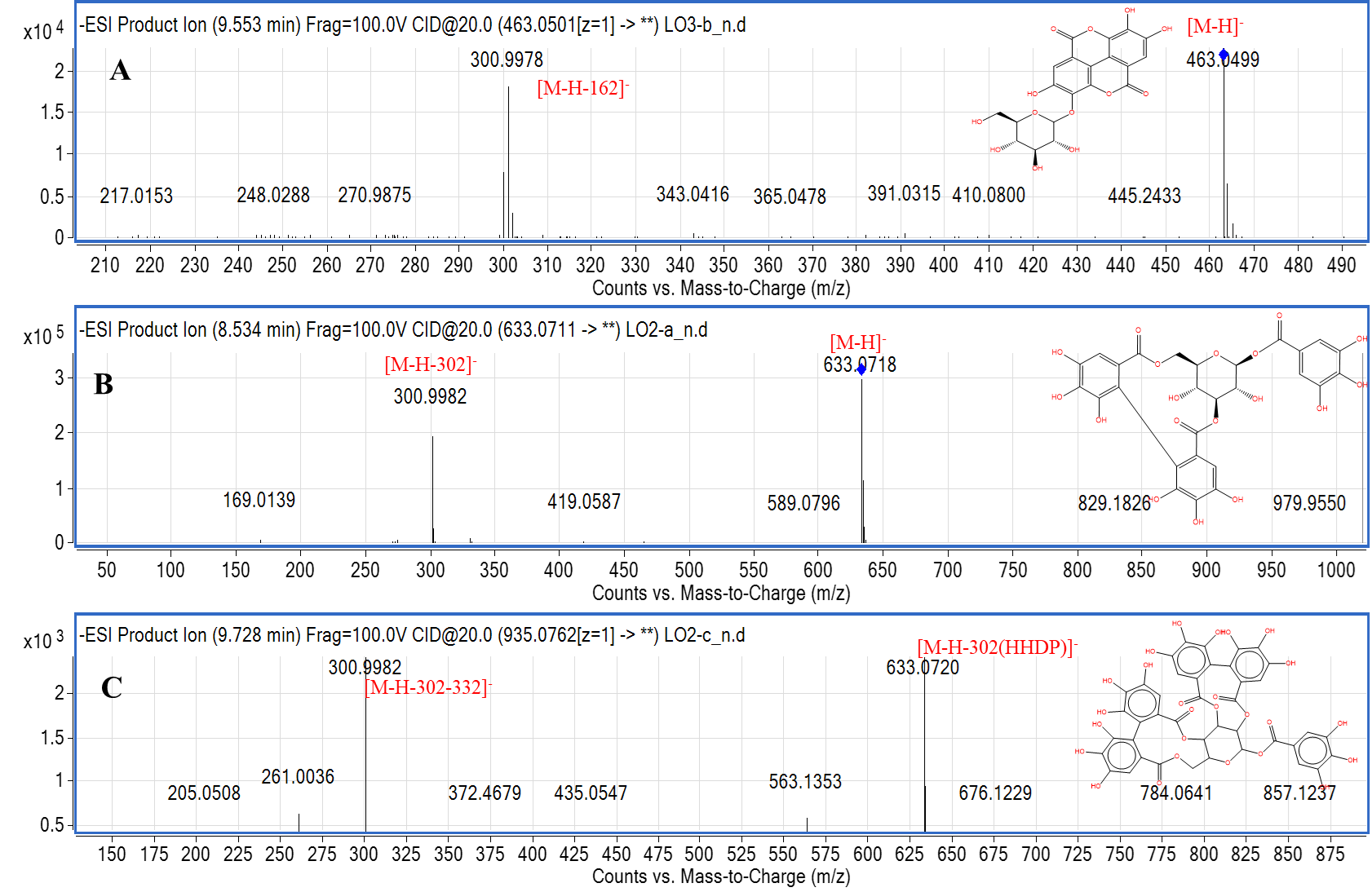


**Suppl. Fig. S5** MS/MS spectra of (A)ellagic hexoside, (B) corilagin, and (C) casuarictin in the negative ion mode.

**Suppl. Fig. S6** Molecular network of flavonoids family in *Nymphaea nouchali* extracts; flower, stem and leaves based on MS/MS in negative mode.

**Suppl. Fig. S8**.MS/MS spectra of (A)patuletin-*O*- galloyl hexoside and(B) patuletin-*O*-hexoside in the positive ion mode.

**Suppl. Fig. S9**. MS/MS of (A) Myricetin-*O*-acetyl rhamnoside;(B) Isorhamnetin-*O*-hexoside in the negative mode.

**Suppl. Fig. S10**.MS/MS spectra of A) Eriodictyol-*O-*hexoside; B) Naringenin-*O*-hexoside in the negative ion mode.

**Suppl. Fig. S11** Molecular family of some alkaloids based on MS/MS in positive mode.

**Suppl. Fig. S12.** MS/MS spectra of malic acid, citric acid.

**Suppl. Fig. S12**. MS/MS spectra, of (A) feruloyl coumaroyl spermidine; (B) tri-coumaroyl spermidine; in the positive ion mode.

**Suppl. Fig. S13**. MS/MS spectra of tryptamine in the positive ion mode.

**Suppl. Fig. S14**. MS/MS spectra of malic acid, citric acid and succinic acid in the negative ion mode.

**Suppl. Fig. S15**. MS/MS spectra of hydroxy hexadecanoic acid and oleamide in the negative mode.

**Suppl. Fig. S16.** MS/MS spectra of (A ) sulfoquinovosyl monoacyl glycerol (18:3),B) PE(18:3/0:0) in the negative ion mode.

**Suppl. Fig. S17**. MS/MS spectra of (A ) Mono galactosyl monoacylglycerol MGMG (18:3) and (B) PA(18:2) in the negative ion mode.
